# Lenvatinib resistance mechanism and potential ways to conquer

**DOI:** 10.3389/fphar.2023.1153991

**Published:** 2023-04-20

**Authors:** Wentao Bo, Yan Chen

**Affiliations:** ^1^ Department of Hepatopancreatobiliary Surgery, Sichuan Clinical Research Center for Cancer, Sichuan Cancer Hospital and Institute, Affiliated Cancer Hospital of University of Electronic Science and Technology of China, Chengdu, China; ^2^ Department of Pharmacy, Sichuan Clinical Research Center for Cancer, Sichuan Cancer Hospital and Institute, Affiliated Cancer Hospital of University of Electronic Science and Technology of China, Chengdu, China

**Keywords:** Lenvatinib, drug resistance, mechanism, pharmacological parameters, hepatocellular carcinoma

## Abstract

Lenvatinib (LVN) has been appoved to treat advanced renal cell carcinoma, differentiated thyroid carcinoma, hepatocellular carcinoma. Further other cancer types also have been tried in pre-clinic and clinic without approvation by FDA. The extensive use of lenvastinib in clinical practice is sufficient to illustrate its important therapeutic role. Although the drug resistance has not arised largely in clinical, the studies focusing on the resistance of LVN increasingly. In order to keep up with the latest progress of resistance caused by LVN, we summerized the latest studies from identify published reports. In this review, we found the latest report about resistance caused by lenvatinib, which were contained the hotspot mechanism such as the epithelial-mesenchymal transition, ferroptosis, RNA modification and so on. The potential ways to conquer the resistance of LVN were embraced by nanotechnology, CRISPR technology and traditional combined strategy. The latest literature review of LVN caused resistance would bring some ways for further study of LVN. We call for more attention to the pharmacological parameters of LVN in clinic, which was rarely and would supply key elements for drug itself in human beings and help to find the resistance target or idea for further study.

## Graphical Abstract

**Figure:**
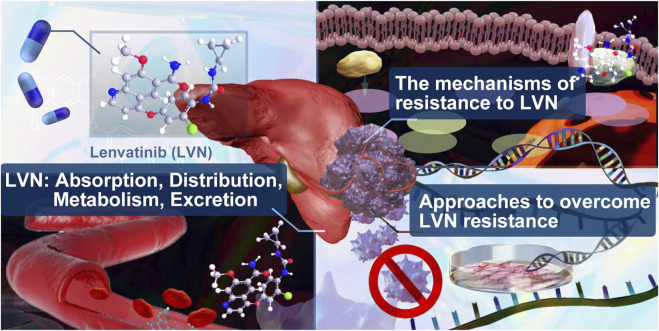
In this review, we summerized the absorption, distribution, metabolism, excretion of lenvatinib in clinic. The lastest mechanism and potential approches to overcome resistance caused by lenvatinib were supplied.

## 1 Introduction

**FIGURE 1:**
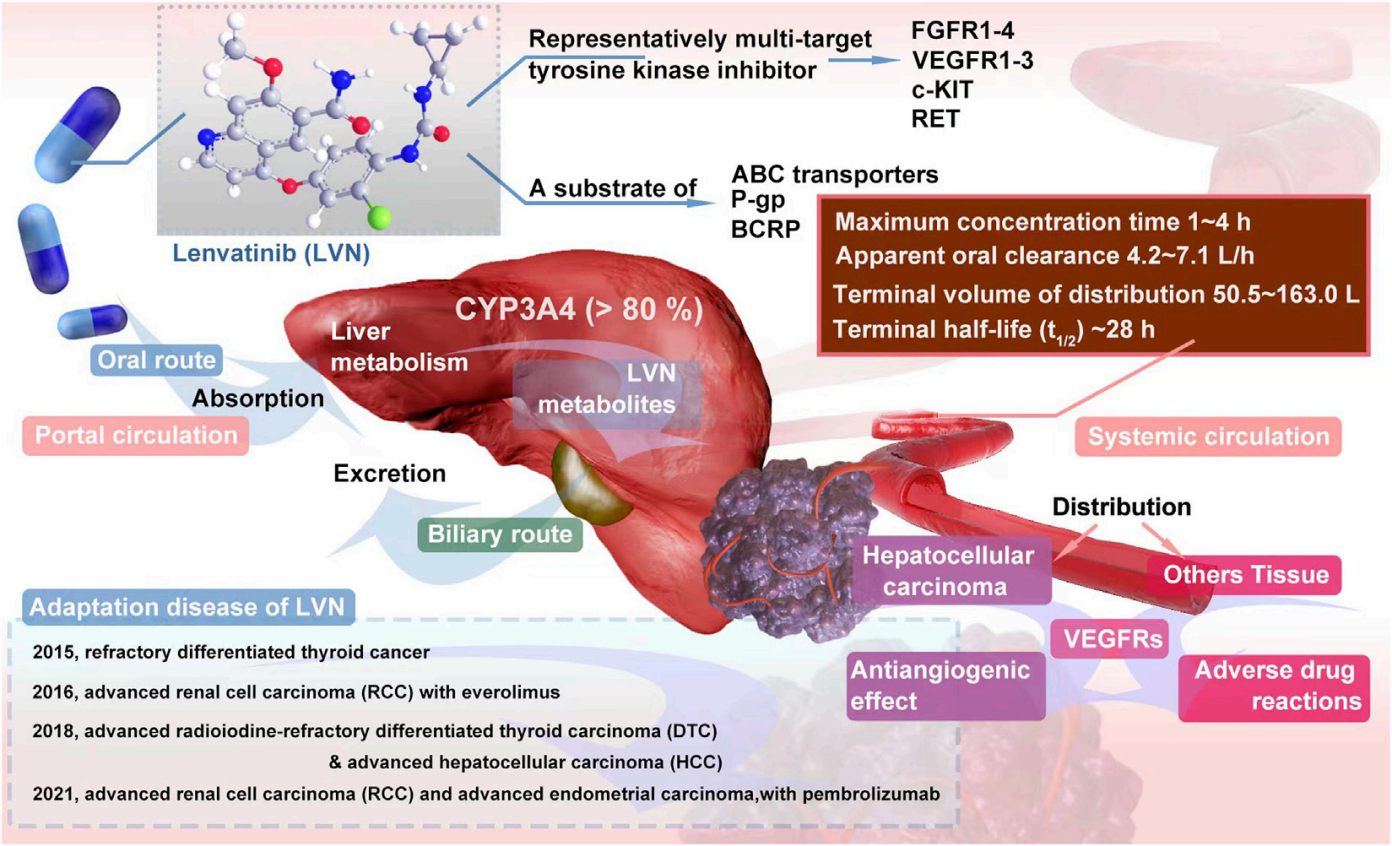
Overview of LVN pharmacodynamics and pharmacokinetics *in vivo* and the time line of Indication approval.

Lenvatinib (LVN) is one of the representatively multi-target tyrosine kinase inhibitor. The target of LVN contains fibroblast growth factor receptors 1-4 (FGFR1-4) (Uehara et al., 2022), vascular endothelial growth factor receptors 1-3 (VEGFR1-3) (Muraishi et al., 2022), stem cell factor receptor (c-KIT) (Chen et al., 2022a) and rearranged during transfection (RET) (Hegde et al., 2020). As an oral tablet or capsule, Food and Drug Administration (FDA) approved LVN for refractory differentiated thyroid cancer in 2015 (Inc E, 2015; FDA, 2016). In the next year, the indication for advanced renal cell carcinoma (RCC) with everolimus was approved (Motzer et al., 2015). In 2018, the usage for advanced radioiodine-refractory differentiated thyroid carcinoma (DTC), which acted as the second-line plan, has been confirmed by FDA (Wirth et al., 2018). In the same year, LVN was approved for the first time as a treatment for hepatocellular carcinoma (HCC). Latestly, in 2021, FDA approved the combination of LVN plus pembrolizumab for advanced renal cell carcinoma (RCC) and advanced endometrial carcinoma (EC) as the first-line treatment regimen of adult patients The timeline of LVN Indication approval was exhibited in the Figure 1. The effective therapy from LVN gains large attention from clinicians. Herein, the question of LVN resistance has naturally become a hot topic for scientists to study, no matter in the cancer types of RCC, HCC or DTC (Hu et al., 2022a; Persano et al., 2022). At present, LVN has achieved good efficacy in the targeted therapy of many cancer types (Su et al., 2022a; Wassermann et al., 2022). However, in cancer types such as liver cancer, once LVN is resistant to drugs, the drug effect of second-line therapy is often poor (Jindal et al., 2019). Therefore, exploring the mechanism of LVN resistance and delaying the occurrence of drug resistance can effectively prolong the life cycle of these patients. Herein, in this article, we summerized the latest resistance mechanisms, meanwhile provided the lastest train of thought to conquer or decrease the resistance. Further, discussion from the scope of drug itself, especially in clinical pharmacological character, to make some effort to enhance the therapeutic outcome of LVN would bring more benefits.

## 2 Clinical pharmacological character of LVN

### 2.1 Clinical absorption, distribution, metabolism, execretion of LVN

As shown in Figure 1, LVN is administered by oral route at doses from 8 to 24 mg per day. The specific dosage was fixed according to which type of cancer and which stage of cancer (Taylor et al., 2022). After oral administration, the absorption rate of LVN, could be reached to 98%–99% with plasma proteins primarily to albumin, and the binding is concentration independent (Ye et al., 2021). A high-fat diet slows the absorption of LVN, but has no significant effect on systemic exposure (Shumaker et al., 2014a).

The maximum concentration of LVN was floated between 1 h and 4 h (Shumaker et al., 2014a) after absorption orally. The terminal half-life (t_1/2_) was about 28 h, which was considered to be taken as once a day regimen (Dubbelman et al., 2012). The apparent oral clearance was ranged from 4.2 L/h to 7.1 L/h, with terminal volume of distribution from 50.5 L to 163.0 L, respectively. According to a large sample size (n = 779) of LVN clinical study data, three-compartment model with linear elimination was reproted (Gupta et al., 2016). The clinical pharmacological character of LVN was displayed in the Table 1.

**TABLE 1:** Clinical pharmacological character of LVN.

Clinical pharmacological character of LVN		
Parameters	Data	Refs
Maximum concentration	1–4 h	Shumaker et al. (2014a)
t_1/2_	28 h	Dubbelman et al. (2012)
Apparent oral clearance rate	4.2 L/h to 7.1L/h	Gupta et al. (2016)
Terminal volume of distribution	50.5 L–163.0 L	Gupta et al. (2016)
Dose-normalized AUC	3710 ng*h/ml	Gupta et al. (2016)

LVN is mainly metabolized by cytochrome P450 (CYP) in the liver. More than 80% was metabolized by CYP3A4 (Gupta et al., 2016). Meanwhile, as a substrate of both ATP-binding cassettes (ABC) transporters, P-glycoprotein (P-gp) and breast cancer resistance protein (BCRP), LVN could be transported by them (Ozeki et al., 2019). One report concluded that ketoconazole could increase the maximum plasma concentration of LVN, while the elimination half-life of LVN was not altered (Shumaker et al., 2015). As we know, ketoconazole is a inhibitor of P-gp and BCRP.

The main metabolites of LVN contains decyclopropylation, demethylation, N-oxidation, and O-dearylation with the help of MS assay (De Mattia et al., 2019). The excretion route of LVN is via the biliary route. There was no accumulation even after multiple daily doses (Boss et al., 2012). What’s more, PK parameters, such as apparent clearance, distribution volumn (Zhang et al., 2022a) of LVN was unaffected by pH-elevating agents (including proton pump inhibitors, antacids, H2 blockers), age (from 18 to 89), race (including while, black, asian, japanese, hispanic and other), and renal function (creatinine clearance) (Gupta et al., 2016) and so on.

### 2.2 Clinical plasma concentration of LVN

The reports about clinical plasma concentration of LVN were insufficient. The report concerned with clinical plasma data also with parameter of PK/PD was supplied by Ikeda M et al. (Ikeda et al., 2016). In this study, 20 patients were enrolled. The C_ss_ of LVN was ranged from 346 to 349 ng/ml of the multiple dose (12 mg daily). Another differentiated cancer of the thyroid SELECT study (n = 260) found that the mean dose-normalized AUC was 3710 ng*h/ml (Gupta et al., 2016). Thirty-two healthy Chinese volunteers were enrolled with 8 mg dosage of LVN per day. The maximum concentration of LVN in the group of CYP3A4*1G/*1G allele carrier subjects was 73.4 ng/ml, which was higher than the group of *1 carrier (53.5 ng/ml). However, the steady state concentration data were not included in this study (Li et al., 2020). Similarly, 40 Japanese patients with thyroid cancer study supplied that pharmacokinetic parameters of LVN were signifcantly infuenced by the carrier of 20230G>A on CYP3A4 (Ozeki et al., 2019). The dose-adjusted C_0_ (ng/mL/mg) was used to denote the concentration of LVN. The specific concentration of LVN was still missing.

The obstacle for LVN clinical monitoring might be related with these reasons. 1) For clinical patients, compliance is very poor. As we known, the time points of PK experiments were very intensive. As claimed by Ikeda M et al. (Ikeda et al., 2016), blood samples were obtained for PK analysis on day 1 (predose and 0.5, 1, 2, 4, 6, 8, and 24 h postdose), day 8 (predose), day 15 (predose and 0.5, 1, 2, 4, 6, 8, and 24 h postdose), and day 22 (predose) of cycle. These type of blood collection for patients must be a great challenge. Herein, the study about the PK parameters of LVN would be usually in the phase I study rather than in normal clinical study. 2) There are no reliable and sufficient research to refer, especially on the relationship between specific concentration of LVN and advers reaction. Hence, the attention about the concentration of LVN was few. If the multi-ethnic and multi-central study focusing on the concentration of LVN could be done, the precision therapy of LVN would be giving huge step forward. Hence, we suggest that more attention on the concentration of LVN in human beings. The real concentration of LVN is the most intuitive indicator of a drug’s effect in the body, which other indirect indicators can not be replaced. The concentration study should be explored first and be considered as a basis for other research.

## 3 Resistance mechanism

The drug resistance is normal dillema among nearly all therapeutic drugs. As the time of therapy prolonging, the cancerous cells could adapt the blocking pathway. Then the drug resistance appeares. The drug resistance mechanisms of LVN included blockage the target of VEGFR, FGFR, PDGFR, KIT and RET, which has been specifically depicted in previous reviews (Al-Salama et al., 2019; Zhao et al., 2020; Mo et al., 2021; Wirth et al., 2022). Because LVN acts on these above-mentioned multiple molecular targets, these types of resistance mechanism are among the first to be studied. Additionally, the cell apoptosis, cell ferroptosis, checkpoint regulation, cytokine overproduction, N6-threonylcarbamoyladenosine modification and so on, emerged latestly. Herein, we summerized the latest resistance studies and cleared up them from primary or acquired resistance about LVN aiming to provides a potential classification and treatment strategy for LVN.

### 3.1 EMT-related resistance

The epithelial-mesenchymal transition (EMT) in cancer cells not only results in metastasis, but also contributes to drug resistance in recent years (Liu et al., 2021; Pan et al., 2021; Kichi et al., 2022). The brief process of EMT could be drew as epithelial cells displaying collapse of cell-cell junctions, then temporaryly transiting to cells with ability of migration (Hardy et al., 2010; Erin et al., 2020). The expression of fibroblast growth factor receptor 1 (FGFR1) has been playing critical role in EMT, which could promote the occurrence of EMT (McNiel and Tsichlis, 2017; Roy Burman et al., 2021).

**FIGURE 2:**
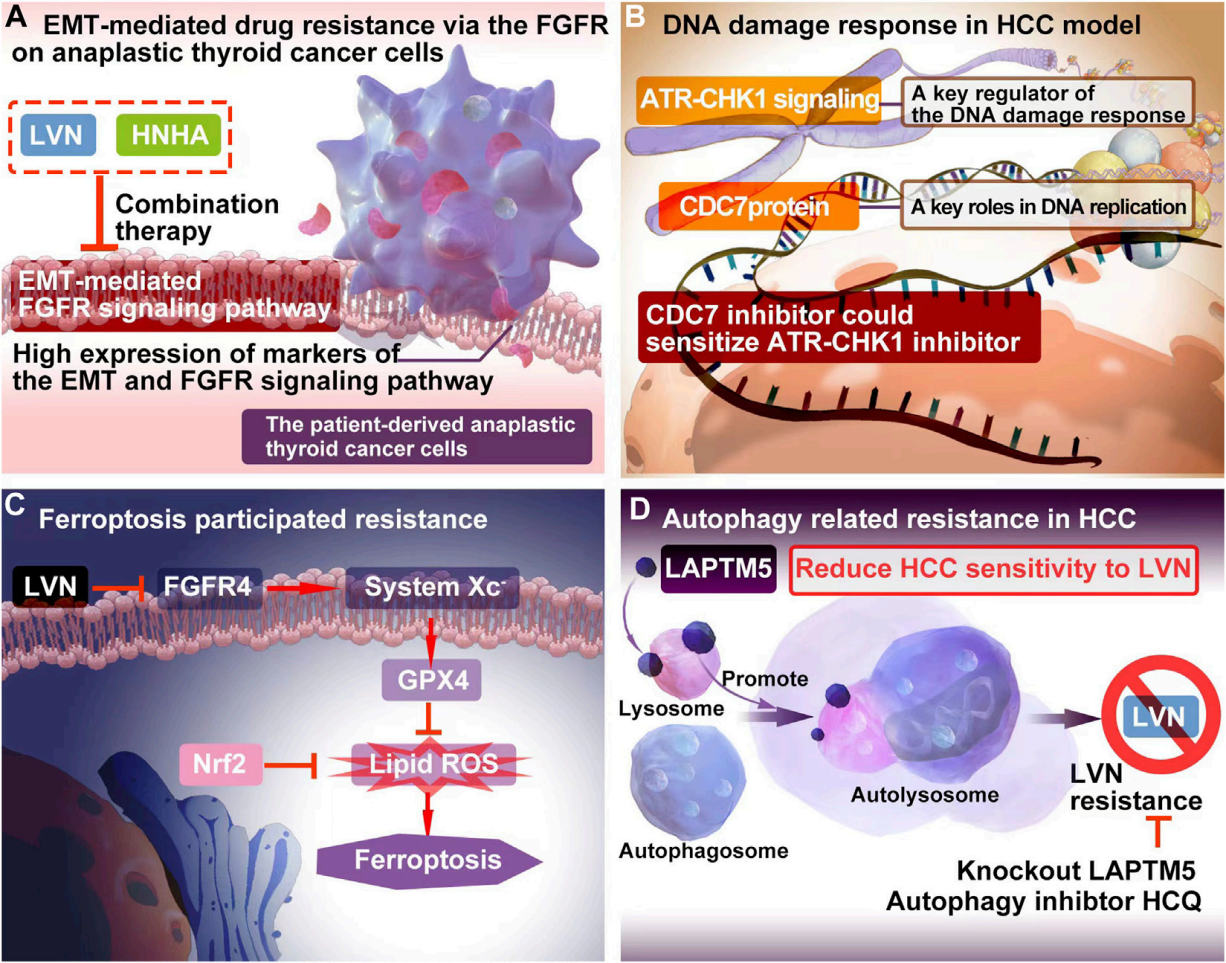
The resistance mechanism focuing on EMT, DNA damage, ferroptosis and autophagy of LVN. **(A)** EMT-mediated drug resistance via the FGFR onanaplastic thyroid cancer cells. **(B)** The combination of DNA damage response regulator with the inhibitor of CDC7 could bring out better therapuetic outcome of LVN in HCC via inhibiting DDR mediated drug resistance. **(C)** LVN involved in inhibition of ferroptosis participating process through blockage of the cystine import activity of GPX4. **(D)** Blocking intrinsic autophagic flux by knockout LAPTM5 or adding autophagy inhibtor, HCQ, could be overcome resistance caused by LVN.

LVN acted as FGFR inhibitor, while sorafenib played role as tyrosine kinase inhibitors. The inhition of FGFR might be potential to decrease EMT. Herein, Lee YS et al. (Lee et al., 2018). included 3 cell models, which included patient-derived PTC cells, patient-derived ATC cells and resistance to sorafenib ATC cells. In this study, sorafenib was acted as a positive control. The treatment regimen was designed as group one for LVN alone, group two for sorafenib alone, group three for LVN with HNHA (histone deacetylase) and group four for sorafenib with HNHA. HNHA is quitely required in TGFβ1 induced EMT. The experimental data exhibited that group three was more effective than other three groups. These findings have implications for ATC treatment by preventing drug resistance in cancer stem cells and further this type of drug resistance belonging to the acquired drug resistance. The potential EMT-related mechansim of LVN was depicted in the Figure 2A.

### 3.2 DNA damage response (DDR) involved resistance

Genomic instability is a key symbol of tumor occurrence, development, metastasis, drug resistance that arises owing to defects in the DNA damage response (DDR) (Pilié et al., 2019; Reisländer et al., 2020). As more and more DDR signaling pathways are discovered, the resistance therapy of DDR supplies an attractive way (Chao et al., 2022). The cell cycle checkpoint kinases CHK1(checkpoint kinase 1) and CHK2 (checkpoint kinase 2) act together on DDR pathways and are immediate targets of ATR (taxia-telangiectasia and Rad3 related) and ATM (ataxia-telangiectasia mutated), respectively (Serra et al., 2022).

Guo Y et al. (Guo et al., 2021a) concluded that ATR-CHK1 signaling would be a therapeutic target for liver cancer. As reported, the role of ATR-CHK1 has been considered as a necessary regulator of the DDR, which especially involved in sensing DNA replication stress (Smith et al., 2020; Khazaaleh et al., 2021; Vazhappilly et al., 2021). Further, this signal could activate oncogene and regulate G1 checkpoint (Khazaaleh et al., 2021). Until now, the inhibitor of ATR and CHK1 exhibited good therapeutic effect on tumor in labortary, while the strategy on clinic still recommended the combination therapy of them.

The cell division cycle 7 (CDC7) protein conferred in S-phase checkpoint and M-phase completion during DNA replication initiation (Montagnoli et al., 2010). The significant different expression of CDC7 in HCC tumor tissues comparision with non-tumor tissues pointed out a beautiful window for HCC treatment (Rojas-Prats et al., 2021). Rojas-Prats E et al. (Rojas-Prats et al., 2021) chose eight human HCC cell lines, which containing Hep3B, SNU398, SNU449, SNU182, Huh7, Huh6, PLC/PRF/5 and HepG2 to verify. In this study, the inhibitor of CDC7 increased DNA replication stress and then sensitized tumor cells to ATR or CHK1 inhibitors, respectively. As a result, the comnbination of ATR and CDC7 inhibitors would be recommened. The DDR involved resistance mechansim of LVN was depicted in the Figure 2B according to the corresponding reports (Guo et al., 2021a), and further this type of drug resistance belonging to the primary drug resistance.

### 3.3 Ferroptosis participated resistance

In the past decade, ferroptosis has been found confering more and more types of tumours (Chen et al., 2021; Lei et al., 2022). Ferroptosis is different from other types of cell death, which containing cell apoptosis, necrosis, and autophagy (Hassannia et al., 2019; Zhang et al., 2022b). The characteristic of ferroptosis is that it’s highly accompanied by the iron-dependent accumulation of lethal lipid reactive oxygen species (ROS) (Huang et al., 2023). The promotion of ferroptosis could significantly improve the killing ability of cancerous cells.

Iseda N et al. (Iseda et al., 2022) found out that LVN could suppress the expression of glutathione peroxidase 4 (GPX4) via decreasing the cystine import activity of GPX4 and eventually leading to the accumulation of lipid ROS. Silencing-FGFR4 suppressed GPX4 expression and increased lipid ROS levels. Further the activation of Nrf2 suppressed ferroptosis similarly by lipid ROS accumulation. Herein, Nrf2 inhibitors could be efficiently with LVN in the near future. The ferroptosis participated resistance mechansim of LVN was depicted in the Figure 2C according to the abovementioned reports (Iseda et al., 2022). Because of ferroptosis was caused by LVN, the resistance would grouped to acquired drug resistance.

### 3.4 Autophagy related resistance

Autophagy is regarded as an adaption way for tumor cells to survival along with long time evolution. Herein, it has been certified to play a double-edged sword in drug resistance (Smith and Macleod, 2019; Yao et al., 2022). On one hand, autophagy participates in the development of drug resistance and protects cancer cells from chemotherapeutics (Gao et al., 2022). On another hand, it kills resistance cancer cells via promoting cell autophagy (Jin et al., 2022). Pan J et al. (Pan et al., 2022) integrated unbiased whole-genome CRISPR-Cas9 screen with database analysis indicated LAPTM5 (lysosomal protein transmembrane 5) as the critical contributor to LVN resistance in HCC. LAPTM5 was located on the membrane of lysosome (Zouali, 2014). LAPTM5 could drastically promote autophagic flux by facilitating autophagososme-lysosome fusion to reduce HCC sensitivity to LVN. Blocking intrinsic autophagic flux by knockout LAPTM5 or adding autophagy inhibtor, HCQ, could be overcome resistance caused by LVN. This finding supplied a potential combination strategy for LVN therapy in clinic.The autophagy participated resistance mechansim of LVN was supplied in the Figure 2D according to the abovementioned reports (Pan et al., 2022). Similarly, autophagy was induced by LVN. This type of resistance could be acted as the acquired drug resistance.

### 3.5 RNA involved in regulating resistance

Long non-coding RNAs (lncRNAs) plays an important role in drug resistance (Wang et al., 2019). Numerous studies supplied that lncRNAs involved in drug resistance through coordinating with microRNAs (miRNAs) and protein-coding mRNAs via influencing transcription, post-transcription and translation (Jiang et al., 2020; Li et al., 2021a). Recently, lncRNAs function has been recommened as competitive endogenous RNAs (ceRNAs), which integrated with miRNAs and adjusting the expression of their downstream target genes (Xuan et al., 2019).

Yu T et al. (Yu et al., 2021) found lnc-RNA MT1JP was upregulated in LVN resistant HCC (LR-HCC) cells, when compared with none resistance HCC cells. The anti-apoptotic protein, namely Bcl-2 like 2 (BCL2L2), could sponge of microRNA-24-3p by MT1JP releasing. The BCL2L2, microRNA-24-3p and MT1JP formed a positive-feedback loop to promote the drug resistance. Wang Y et al. (Wang et al., 2022a) reported a novel lncRNA, AC026401.3, which promoted sorafenib and LVN resistance in HCC cell lines. AC026401.3 interacted with OCT1 and promoted the recruitment of OCT1 to the promoter region of E2F2. Consequently, it upregulated the expression of the transcription factor E2F2. Lastly, LVN resistance in HCC was appeared. The MT1JP was screened from LR-HCC cells. The mechanism of lnc-RNA MT1JP was derivated from acquired drug resistance.

**FIGURE 3:**
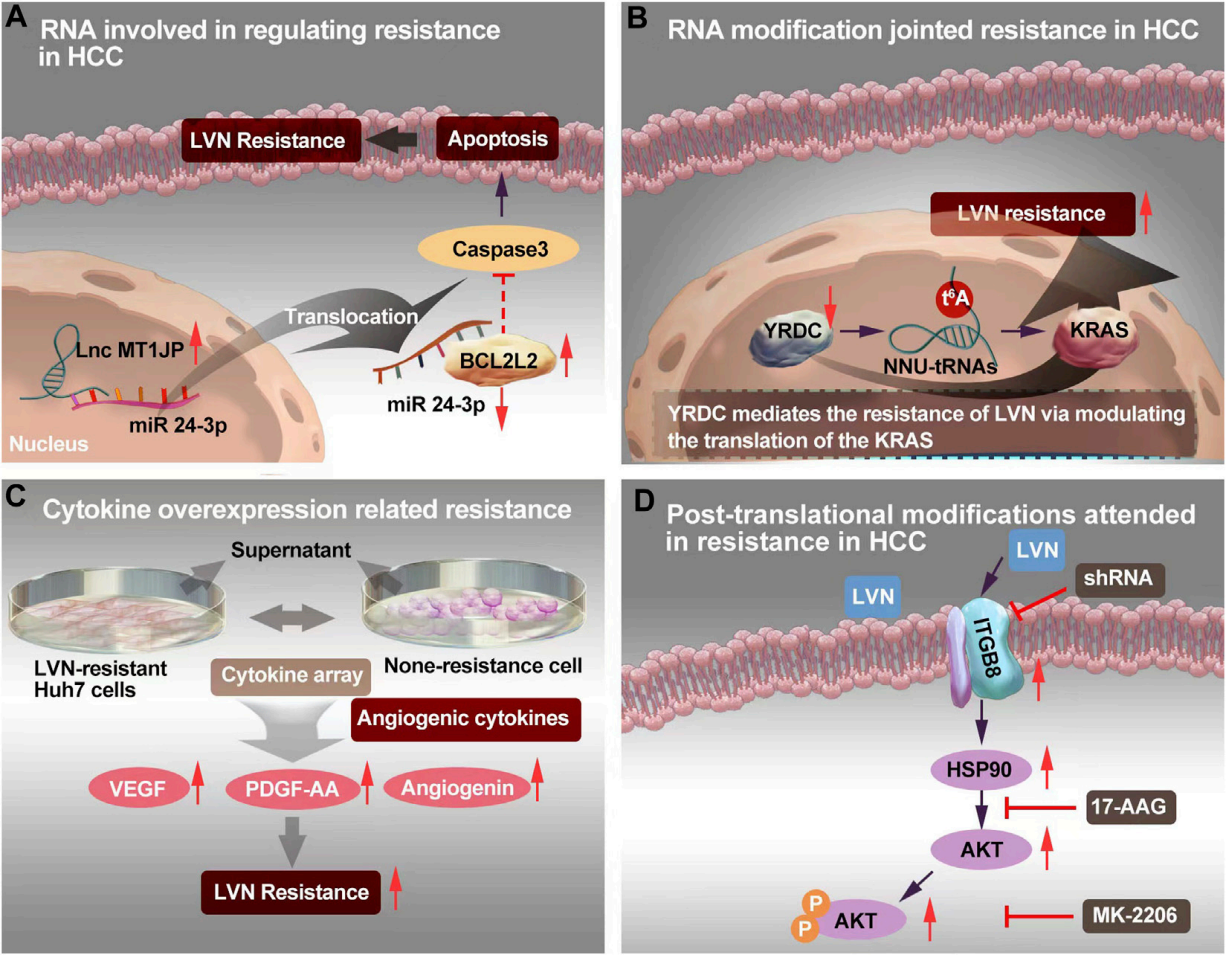
The resistance mechanism focuing on RNA, RNA modification, cytokine and post-translational modification of LVN. **(A)**Sponging of microRNA-24-3p by MT1JP released BCL2L2, thereby forming a positive-feedback loop to realize LVN resistance. **(B)**YRDC knockdown showed decreased sensiticity to LVN via t6A modification. **(C)** Angiogenic cytokine overproduction confered in LVN-resistance in HCC and thereby contribute to tumor angiogenesis to increase the occurence of resistance. **(D)** Up-regulated ITGB8 in LVN resistance HCC cells leading to AKT’s ubiquitination to achieve resistance.

Xu X et al. (Xu et al., 2022) reported that the overexpression and activation of c-Met participating in LVN resistance human HCC cell line, Huh7 and SMMC-7721 cell line. The upstream mechanisms was found related with miRNA-128-3p. The miR-128-3p regulated the expression of c-Met negatively and it was lower expression in LVN resistance cell line rather than in none resistance cell line. The miR-128-3p/c-Met axis was found out that it adjusted proliferation and apoptosis-related signaling pathways in LVN resistance cell line to realize and promote resistance. RNA involved in regulating resistance in HCC was supplied in the Figure 3A according to the report of Yu T et al. (Yu et al., 2021). The function of miRNA-128-3p would also be regarded as acquired drug resistance.

### 3.6 RNA modification jointed resistance

RNA modification incorporated with severing and base modification was remained as potential targets of cancer accurate therapy (Barbieri and Kouzarides, 2020; Liu et al., 2023). N6-threonylcarbamoyladenosine (t6A) and its derivatives are universally conserved modified nucleosides and belongs to the most common modification of tRNA modification (Wang et al., 2022b). This type of modification has multiple roles of tRNAs in decoding and protein synthesis (Matuszewski et al., 2017).

YRDC is highly conserved and derived from *E. coli* to *Homo sapiens* (Jia et al., 2002). YRDC/Sua5 family confers in the t6A biosynthesis of tRNA. This family has been proved promotion the resistance to EGFR-TKI pathway (Shi et al., 2022). The t6A modification mainly decoding ANN codons is one of the 15 universally conserved modification (Cantara et al., 2011). The t6A modification could reinforce the codon-anticodon interaction and improve translational fidelity by the ribosomes (Su et al., 2022b). YRDC/Sua5 could influence the protein translation at the level of codon recognition (Yarian et al., 2000). Further, YRDC promoted the proliferation of HCC cells via activating the RAS/RAF/MEK/ERK signal pathway. And this signal axi was the primary pathway of LVN in the treatment of HCC (Shi et al., 2022). Herein, team of Guo J et al. (Guo et al., 2021b) made brave speculation about the role of YRDC. Guo J et al. (Guo et al., 2021b) demonstrated that YRDC knockdown’s Huh7 cells exhibted less sensiticity to LVN comparision with parental cells. Furthermore, the expression of YRDC was decreased in a time dependent manner by LVN. This phenonmen could explain the occurence of LVN resistance in clinic with the prolong therapeutic time. Consequently, the effect of tRNA with low t6A modification levels could dramatically reduce the translation of the KRAS *in vitro* translation system and mediate resistance of LVN. The YRDC was verified from cells without LVN longterm induced. Herein, it might be probably suggested to regarded as the primary drug resistance. The potential mechansim was exhibited in Figure 3B.

### 3.7 Cytokine overexpression related resistance

Cytokine plays “telephone” roles in cell to cell, or organelle to cell. The cytokine-mediated signaling networks, which is relevant to tumor progression, metastasis and resistance, and supply new window into the mechanistic details for novel therapeutics for cancer (Bhat et al., 2022; Gandhi et al., 2022). In terms of cytokines, Ao J et al. (Ao et al., 2021) isolated supernatant derived from LVN-resistant Huh7 cells to comparing with none-resistance cell. Totally, 105 different antibodies were identified via a cytokine array. Among them, 16 cytokines were overproduced. Only three angiogenic cytokines: VEGF, platelet-derived growth factor-AA (PDGF-AA), and angiogenin, were dramatically different compared with the control. Of importance, the supernatant of cell culture from LVN-resistant Huh7 cells exhibited the ability of acceleration tube formation by HUVECs. These findings indicated that angiogenic cytokine overproduction confered in LVN-resistance in HCC and thereby contribute to tumor angiogenesis to increase the occurence of acquired drug resistance. The potential mechansim was exhibited in Figure 3C.

### 3.8 Post-translational modifications attended in resistance

Post-translational modifications, which included phosphorylation (Melo-Braga et al., 2021), acetylation (Narita et al., 2019), methylation (Kalinina and Novichkova, 2021), S-nitrosylation (Paakinaho et al., 2021), SUMOylation (Zittlau et al., 2022) and ubiquitylation (Chen et al., 2020), are critical for protein function and interaction with RNA, DNA or other key signal molecules. Post-translational modification will be resulted in conformational in protein structure, biological function of proteins, and signature of metabolic transformations changes (Tikhonov et al., 2021).

Hou W et al. (Hou et al., 2022) chose two LVN resistant HCC cell lines and consequently screened integrin subunit beta 8 (ITGB8) as a critical contributor to LVN acquired drug resistance in HCC cell lines. In this study, ITGB8 was related with phosphorylation of HSP90. The phosphorylation of HSP90 could lead to the ubiquitination of AKT and then degrade of AKT. The potential mechansim was depicted in Figure 3D.

## 4 Potential ways to conquer LVN’s resistance

### 4.1 Active target therapy to enhance the efficacy and prolong the time to onset of resistance

The emergency of nanotechnology, which is based on passive or active targeting drug delivery systems, is as the most promising strategy for cancer therapy owing to the size and surface properties of nanomedicines could contribute to the improvement of pharmacokinetic and pharmacodynamic for intracellular delivery of anti-cancer drugs (Azizi et al., 2022; Hao et al., 2022; Magne et al., 2022). Intrahepatic cholangiocarcinoma (ICC) is a primary hepatocellular carcinoma that originates from the region of the intrahepatic bileduct epithelium to the heringian duct epithelium. Zhouyu Ning et al. (Ning et al., 2022) successfully constructed H-MnO_2_-FA nanoparticles, which aimed target ICC and delivered LVN actively. This nano drug delivery system demonstrated effectively inhibition rate of cell proliferation and cell apoptosis rate in 9810 cells. Furthermore, the inner mechansim of resistance conferred in the activation of Raf1-MEK1/2- ERK1/2 signaling pathway.

Xu Q et al. (Xu et al., 2021) constructed LVN with copper sulfide nanocrystals (Cu_2-x_S NCs) via the carrier of poly (D,L-lactide-co-glycolide) (PLGA) to implementation the outstanding photothermal properties in the near-infrared-II (NIR-II) zone to treat HCC. This nanocrystal system exhibited excellent antitumor effect even without recurrence and suppressed the expression of P-glycoprotein (P-gp) protein and MDR relevant protein no matter in MHCC97H cell model but also in MHCC97H derived subcutaneous tumor mice model. The average masses of tumors of negative control (0.85 g) was nearly 42.5 fold compared with group of LVN-loaded nanoparticle with NIR laser (0.02 g). This chemo-photothermal with nano-technology supplied a new view to conquer LVN resistance from the scope of dramatically enhancing the effect of LVN.

Giammona, G. et al. (Giammona et al., 2022) designed NIR-responsive hybrid nanocomposites. This nanocomposites was consisting of an amphiphilic polyhydroxyaspartamide-based graft copolymer (PHEA-g-BIB-pButMA-g-PEG-GAL), which embedd hydrophobic gold nanorods simultaneously. This hybrid nanocomposite aimed at a galactose-mediated smart composite nanosystem to achieve an efficient loading of sorafenib and LVN onto asialoglycoprotein receptor overexpressing hepatic cells via NIR-light stimulation. This innovative approach has the advantage to be smart candidates for selective dual-mode therapy and nanotherapy of hepatocarcinoma.

### 4.2 Combined therapy to promote sensitization of LVN

A rational design of combined therapy could dramatically decrease the resistance, no matter in clinic or in experiment (Dai et al., 2022; Liu et al., 2022; Malik et al., 2022; Wu et al., 2022).

Che jui Yang et al. (Yang et al., 2022) reported amentoflavone sensitizing the therapuetic of LVN on HCC. Amentoflavone was a flavonoid isolated from many natural plants (Ullah et al., 2020). Their results indicated that the combination of amentoflavone and LVN further down-regulated c-FLIP, MCL-1, XIAP, and cyclin D1 expression compared to treatment with amentoflavone or LVN alone. Myeloid cell leukemia-1 (Sulkshane and Teni, 2022), XIAP (Hanifeh and Ataei, 2022)and c-FLIP (Ivanisenko et al., 2020) are antiapoptotic proteins that mediate the resistance of tumor cells to anticancer agents through the prevention of apoptosis. Increased levels of antiapoptotic proteins were also shown to be associated with poor therapeutic outcomes in patients with HCC. The mechanim of amentoflavone combined with LVN was to reduct of AKT and ERK phosphorylation to increase the cell apoptosis.

Xi Su et al. (Su et al., 2020) performed a combination mode between LVN and doxorubicin in treating anaplastic thyroid cancer cell lines and xenograft model to verify the capacity of delivering them simultaneously. The combination therapy of LVN and doxorubicin exhibited dramatically inhibition effect on tumor growth, and induction cell apoptosis and cell cycle arrest as compared to lenvatinib or doxorubicin alone on ATC. As the study deeply, LVN could enhance the energy deficiency in mitosis. Doxorubicin could damage DNA (Shetake et al., 2022). Herein, the combined group demonstrated the higher capacity of DNA damage via mitogen-activated protein kinase (MAPK) pathway. The MAPK pathway has been reading of involving in DNA repair especially in response to DNA damage (Maresca et al., 2022). However, further investigations into the detailed molecular mechanisms are still needed.

Sun D et al. (Sun et al., 2022) made a novel model for LVN resistance HCC. They co-administration of elacridar with LVN or gefitinib. The elacridar is a dual multidrug resistance protein 1 (MDR1) and breast cancer resistance protein (BCRP) inhibitor, and widely used in cancer resistance research (Omori et al., 2022). Elacridar (Goutal et al., 2018), a third-generation MDR1 inhibitor, which could enhance therapeutic efficacy in multiple diseases by blocking drug efflux, such as Alzheimer’s disease (Abdallah et al., 2021), chronic Myeloid Leukemia (Alves et al., 2022), and so on. LVN is a substrate of MDR1. The inhibitor of MDR1, such as rifampicin or ketoconazole, could dramatically increase plasma concentration of LVN in healthy adults (Shumaker et al., 2014b; Shumaker et al., 2015). The combination of LVN with elacridar would inhibit LVN efflux by decreasing MDR1 and BCRP efflux pumps. As a result, the scientists verified these theoretical combined mode via activating EGFR, MEK/ERK, and PI3K/AKT pathways. The MDR1 and BCRP transporters were markedly decrease after the combined therapy.

Nakagawa T et al. (Nakagawa et al., 2014) found that combination of LVN and golvatinib could dramatically decrease the hepatocyte growth factor (HGF)-induced resistance via decreasing tumor vessel density in four HCC xenograft models (Gherardi et al., 2012). HGF is a 90 kDa secretory protein with the function of activating intracellular signal transduction (Vimalraj, 2022). Met receptor tyrosine kinase is the sole receptor of HGF. The function of HGF/c-Met signaling pathway included cell proliferation, migration, metastasis and resistance (Barzaman et al., 2022). In this study, as neither LVN nor golvatinib exhibited a direct antiproliferative effect on the HGF-producing cancerous cell lines (SEKI, IM95m, KP-4, and A2780). Further, they illustrated that the VEGF and HGF cooperated to promote tumor angiogenesis, which produced by these types of cancer cells. To sum up, angiogenesis was relatively easier in resistance to single treatment with VEGFR inhibitor, but much more rare in combined treatment with LVN and golvatinib.

He X et al. (He et al., 2022) established LVN-resistant Hep3B cells by long-term exposure to LVN within 2 months. They everified that the activities of EGFR and insulin-like growth factor 1 receptor (IGF1R)/insulin receptor (INSR) were signifcantly increased in resistance cells, whereas the activities of other phospho‐receptor tyrosine kinases were unchanged. Erlotinib, a EGFR inhibitor aiming for non-small cell lung cancer, was found to be participated in the combined therapy with LVN (Zhang et al., 2022c). Erlotinib downregulated abnormally activated ERK and restored the sensitivity of LVN in the resistance cell line. LVN resistance was along with aberrant cholesterol metabolism and activation of lipid raft. Similarly, Hu B et al. (Hu et al., 2022b) verified that ABCB1 could be activated by EGFR in a lipid raft-dependent manner, which significantly improved the exocytosis of LVN to induce resistance. Similarly, clinical samples of HCC displayed a positive correlation between the activation of the EGFR-STAT3-ABCB1 axi and LVN response. Hu B et al. (Hu et al., 2022b) chose Erlotinib, which was demonstrated inhibition on ABCB1, to suppress LVN exocytosis. This synergistic treatment between LVN and erlotinib illustrated a significant antitumor effect on HCC both *in vitro* and *in vivo*.

### 4.3 Blockage the resistance targets

Taken into account of the reports about the LVN resistance, strategies on blockage or knockout, knochdwon the targets supplied the simplest way to conquer resistance. In HCC, CSCs plays the role not only initiating tumor development, inducing tumor progression but also modulating chemotherapy resistance (Fang et al., 2022; Mayani et al., 2022; Liao et al., 2023). Herein, the aim of targeting therapy to eradicate CSCs displays the potential to hinder HCC progression. CD73 is a famous surface marker for recognition mesenchymal stem cells (Bao and Xie, 2022). Ma XL et al. (Ma et al., 2020) concluded that CD73 should be a critical regulator contributing to resistance of LVN. Targeting and purging of CD73^+^ cells is a hopeful strategy for overcoming LVN resistance. AKT participant signaling is a major process for maintaining CSC traits according to accumulating evidence, especially in HCC (Ji and Wang, 2012). SOX9 is a crucial transcription factor for adjusting high mobility group box DNA binding and domains in transactivation (Suryo Rahmanto et al., 2016). Meanwhile, SOX9 has been verified to own stemness characteristics in HCC. Herein, in the study reported from Ma XL et al. (Ma et al., 2020), CD73 switched to control the ubiquitination of SOX9 and the SOX9 was degradated by proteasome via inhibiting GSK3β by activating AKT signaling. Khan HY et al. (Khan et al., 2019) examined LVN resistance anaplastic thyroid cancer cells, namely 8505C cells. With the occurence of resistance, nuclear exporter protein exportin 1 (XPO1) and Rho GTPase effector p21 activated kinases (PAK) was activated along with the change toward mesenchymal morphology. No matter XPO1 or PAK4 inhibitors, when combined with LVN, demonstrated superior anti-tumor activity in 8505C cells inoculated sub-cutaneous xenograft. Further, blockage the XPO1 and PAK4 could increase the sensitivity of the 8505C cells to LVN. Tan Boon Toh et al. (Toh et al., 2020) found that activated Stat3 played an important role in regulating the self-renewing. The side population (SP) (Nayak et al., 2022) and CD44 (Primeaux et al., 2022) were the surface markers from cancerous cells alonging with stemness properties, which could be sorted by flow cytometry. Using ruxolitinib, a Jak/Stat inhibitor, could dramatically decrease p-Stat3 and the number of HCC stem cells. Blockage Jak/Stat axi might be another way to overcome LVN resistance.

The elevation levels of ROS could be as a huge risk factor for the development of PTC in patients with Hashimoto thyroiditis (di Masi et al., 2022; Yi et al., 2015). What’s more, with the increasing aerobic glycolysis, the prognosis of follicular thyroid cancer portends an unfavorable result (Liu et al., 2019). Herein, the glycolytic activity both of ROS level would be proposed as a factor that cannot be ignored for the development of PTC (Sahoo et al., 2016). The prodcing of ROS from different cells was highly related with enzymatic systems, which included NADPH oxidases (NOXs), mitochondrial electron transport chain and so on (Weyemi et al., 2010; Ameziane-El-Hassani et al., 2016). Among the NOXs, NOX4 was the most important one and has been demonstrated to higher expression in PTC (Tang et al., 2018). Tang P et al. (Tang et al., 2022) verified that NOX4 could be used as a glycolytic regulator via ROS among the condition of hypoxia. After the serum-starved conditions via ROS, LVN could be induced glycolysis. With the help of an inhibitor NOX4 (Jiang et al., 2022),GLX351322, LVN’s resistance has been decreased via NOX4 or NOX4-derived ROS. Further, the tumor microenvironments of PTC cells was correspondingly changed. These findings highlight NOX4 and NOX4-derived ROS as a potential therapeutic target in resistance to PTC.

Interferon regulatory factors (IRFs) are belonged to vital nuclear transcription factors, which are consisted of nine members (IRF1-9) in mammals (Li et al., 2021b). The function of IRF proteins sometimes accompanied by tumorous cell proliferation, tumorigenesis, lymphocyte differentiation, regulating immune response, and the develpment of hematopoietic stem cells. Yarong Guo et al. (Guo et al., 2021c) illustrated that IRF2 could promote proliferation, inhibite apoptosis, and increase LVN resistance of HCC cells. Knockout IRF2 could decrease the expression of beta-catenin, while overexpressing IRF2 could increase the expression of beta-catenin. Inhibiting beta-catenin could reverse LVN resistance and targeting IRF2 could improve the therapeutic effect of LVN on HCC.

### 4.4 Basing on new technologies to discover new resistance conferred target

The CRISPR/Cas system is one of the most powerful tools for gene editing most recently (Chen et al., 2022b). Shanzhou Huang et al. (Huang et al., 2022a) verified six genes that were associated with LVN resistance in HCC, which containing DHDH, DUSP4, CCBL1, CNTN2, NOS3 and TNF. After qPCR and western blot verification, the dual specificity phosphatase 4 (DUSP4) coming to light no matter in mRNA level or in protein levels was significantly decreased in LVN resistant HCC cells. The knockout of DUSP4 could improve the survival rate, cell proliferation and migration rate of HCC cells. What’s more, the resistance induced by LVN could be blockage by MEK inhibitor, selumetinib, in the DUSP4 deficiency cell line. The phosphorylation of MEK and activation of ERK caused by DUSP4 deficiency were the integral element for LVN resistance. From the scope of clinical tumor tissues, DUSP4 deficiency was also highly correlated with HCC prognosis and response to LVN. DUSP4 belongs to a member of the dual specificity protein phosphatase subfamily. This type of family takes part in the inactivation of MAPK cascade (Chen et al., 2019). Studies pointed out that higher expression of DUSP4 could be discovered in more aggressive cancers, meanwhile the lower expression or knockout of DUSP4 would promote tumor development and progression in colorectal cancer and glioblastoma (Xue et al., 2018). Herein, the role of DUSP4 could be as a tumor suppressor.

The assay for first genome-wide CRISPR/Cas9-based screening on sorafenib-treated HCC cells was carried out by Zheng A et al. (Zheng et al., 2019), which aimed of identifing essential genes for acquired sorafenib resistance in HCC. In this study, LVN was considered the positive drug model. Among numerous significant difference genes, KEAP1 was remained as the top candidate one. The disruption of KEAP1 counteracted of increasing the resistance of regorafenib (another drug for HCC treatment), and decreasing cell viability and increasing of ROS by LVN. So KEAP1 could influence the resistance induced by sorafenib, lenvatinib, and regorafenib, respectively. Consequently, inner mechanism was found out that Nrf2 conferred in. Specifically, up-regulation of Nrf2 would increase ROS levels and counteracted with KEAP1. Nrf2 also belongs to one of neuclear transfaction fator family (Tsuchida et al., 2017). It was shown in a follow-up experiment that KEAP1/Nrf2 pathway not only involved in the initial treatment stages of primary tumors, but also in later stages of acquired resistance.

Similarly, using CRISPR technolpogy, the HCC driver genes which joint in TKI, were sifted by Myojin Y et al. (Myojin et al., 2021). ST6GAL1 was selected and verified in human HCC cell lines. The ST6GAL1 in serum sample were positively correlated with expression of tumor FGF19 in surgically-resected HCC patients.

Circular RNAs (circRNAs) is a research hotspot in recent years, which displays the function of improve development and progression of various types of cancers (Dashtaki and Ghasemi, 2022; Cao et al, 2022). The circRNA mediator complex subunit 27 (circMED27; circBase: hsa_circ_0006825), which is derived from back-splicing of MED27 mRNA, and islocated on chromosome 9q34.13. Zhang P et al. (Zhang et al., 2021). Demonstrated that circMED27 was postively correlated with the bad prognosis of HCC patients and was significantly overexpression in HCC tissues. More importantly, circMED27 upregulated ubiquitin-specific peptidase 28 expression to revert LVN’s resistance via sponging miR-655-3p. Therefore, knockout circMED27 could be a simple way to derease resistance. The circMED27 itself could be consider as a molecular biomarker for LVN-sensitivity predication and also be a meaningful target for HCC patients.

**FIGURE 4:**
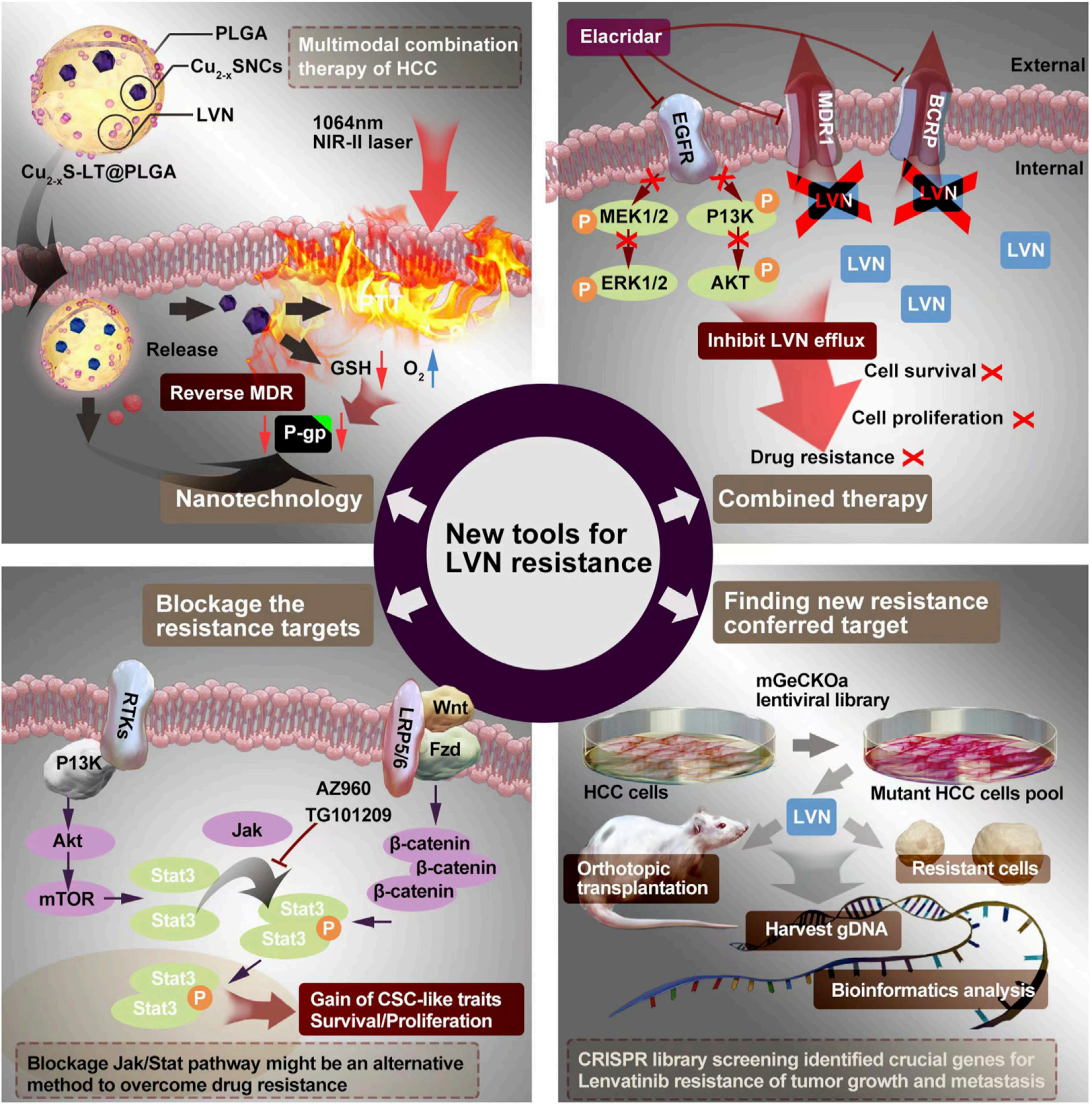
The potential ways to conquer LVN’s resistance containing the nanotechnology, combination therapy, blockage the resistance target and finding new target.

Nowadays, a critical point is the perspective of proteomic analysis suppling the importantly significant proteins no matter in cells or in biofluid (Jia et al., 2022; Riccardi et al., 2022; Yuan et al., 2022). It plays an important role in biomarker discovery, especially in cancer progression, metastasis, and resistance (Riccardi et al., 2022). Huang M et al. (Huang et al., 2022b) performed unbiased proteomic screening of parental and LVN-induced resistant HCC cells (PLC/PRF/5, Hep3B, and Huh7) and discovered two important N7-methylguanosine (m7G) tRNA methyltransferase complex components, which influenced the function related with EGFR translation. Both of the methyltransferase-like protein-1 (METTL1) and WD repeat domain four protein (WDR4) were significantly upregulated in LVN-resistant HCC cells. Depletion of METTL1 decreased the abundance of tRNA m7G modification and restored the abilities to resist LVN-induced cell death. Further, *in vivo* tumor model, lower expression of METTL1 was correlated with lower EGFR expression. This result confirmed that METTL1 could influence the expression level of EGFR. The ways to conquer LVN’s resistance was exhibited in the Figure 4.

## 5 Conclusion and remarks

Untill now, the studies focusing on resistance caused by LVN gains more and more attention, and the resistance targets of LVN in the treatment of HCC were also comprehensively summerized by some scientists (Guo et al., 2022). In this manuscript, we not only

Focused on the resistance mechanism of LVN in the treatment of HCC, but also in RCC, EC, and DTC. The drug resistance mechanisms included EMT, DNA damage, ferroptosis, RNA modification, cytokine overexpression, translational modification and LVN′ self target signal pathway. Basing on the reported resistance mechanisms, the strategies focusing on nanotherapy, combined therapy, blockage new resistance target and digging out unknown functional target via CRISPR or proteomic technologies. It is really appreciated that research on the mechanisms of LVN resistance is so rapid, which will supply sufficient time for its usage applicated in clinic. However, we recommend that more attention on the pharmacological studies about the LVN in human beings, which are rarely in resistance study. The biofluid of LVN in human beings could bring much more information about the intermediary metabolism substance, metabolites, and antimetabolites. When combined with the LVN’s pharmacological characters of circulation in the body, it might provided a “panoramic view” of LVN resistance.

## References

[B1] Abdallah I. M. Al-Shami K. M. Yang E. Kaddoumi A. (2021). Blood-brain barrier disruption increases amyloid-related pathology in TgSwDI mice. Int. J. Mol. Sci. 22 (3), 1231. 10.3390/ijms22031231 33513818 PMC7865722

[B2] Al-Salama Z. T. Syed Y. Y. Scott L. J. (2019). Lenvatinib: A review in hepatocellular carcinoma. Drugs 79 (6), 665–674. 10.1007/s40265-019-01116-x 30993651

[B3] Alves R. Gonçalves A. C. Jorge J. Almeida A. M. Sarmento-Ribeiro A. B. (2022). Combination of elacridar with imatinib modulates resistance associated with drug efflux transporters in chronic myeloid leukemia. Biomedicines 10 (5), 1158. 10.3390/biomedicines10051158 35625893 PMC9138473

[B4] Ameziane-El-Hassani R. Schlumberger M. Dupuy C. (2016). NADPH oxidases: New actors in thyroid cancer? Nat. Rev. Endocrinol. 12 (8), 485–494. 10.1038/nrendo.2016.64 27174022

[B5] Ao J. Chiba T. Shibata S. Kurosugi A. Qiang N. Ma Y. (2021). Acquisition of mesenchymal-like phenotypes and overproduction of angiogenic factors in lenvatinib-resistant hepatocellular carcinoma cells. Biochem. Biophys. Res. Commun. 549, 171–178. 10.1016/j.bbrc.2021.02.097 33676186

[B6] Azizi M. Shahgolzari M. Fathi-Karkan S. Ghasemi M. Samadian H. (2022). Multifunctional plant virus nanoparticles: An emerging strategy for therapy of cancer. Wiley Interdiscip. Rev. Nanomed Nanobiotechnol, e1872. 10.1002/wnan.1872 36450366

[B7] Bao X. Xie L. (2022). Targeting purinergic pathway to enhance radiotherapy-induced immunogenic cancer cell death. J. Exp. Clin. Cancer Res. 41 (1), 222. 10.1186/s13046-022-02430-1 35836249 PMC9284706

[B8] Barbieri I. Kouzarides T. (2020). Role of RNA modifications in cancer. Nat. Rev. Cancer 20 (6), 303–322. 10.1038/s41568-020-0253-2 32300195

[B9] Barzaman K. Vafaei R. Samadi M. Kazemi M. H. Hosseinzadeh A. Merikhian P. (2022). Anti-cancer therapeutic strategies based on HGF/MET, EpCAM, and tumor-stromal cross talk. Cancer Cell Int. 22 (1), 259. 10.1186/s12935-022-02658-z 35986321 PMC9389806

[B10] Bhat A. A. Nisar S. Singh M. Ashraf B. Masoodi T. Prasad C. P. (2022). Cytokine- and chemokine-induced inflammatory colorectal tumor microenvironment: Emerging avenue for targeted therapy. Cancer Commun. (Lond) 42 (8), 689–715. 10.1002/cac2.12295 35791509 PMC9395317

[B11] Boss D. S. Glen H. Beijnen J. H. Keesen M. Morrison R. Tait B. (2012). A phase I study of E7080, a multitargeted tyrosine kinase inhibitor, in patients with advanced solid tumours. Br. J. Cancer 106 (10), 1598–1604. 10.1038/bjc.2012.154 22516948 PMC3349182

[B12] Cantara W. A. Crain P. F. Rozenski J. McCloskey J. A. Harris K. A. Zhang X. (2011). The RNA modification database, RNAMDB: 2011 update. Nucleic Acids Res. 39 (1), D195–D201. 10.1093/nar/gkq1028 21071406 PMC3013656

[B13] Cao C. Wang Y. Wu X. Li Z. Guo J. Sun W. (2022). The roles and mechanisms of circular RNAs related to mTOR in cancers. J. Clin. Lab. Anal. 25, e24783. 10.1002/jcla.24783 PMC9757007 36426933

[B14] Chao Y. Y. Huang B. M. Peng I. C. Lee P. R. Lai Y. S. Chiu W. T. (2022). ATM- and ATR-induced primary ciliogenesis promotes cisplatin resistance in pancreatic ductal adenocarcinoma. J. Cell Physiol. 237, 4487–4503. 10.1002/jcp.30898 36251015

[B15] Chen E. Lin-Shiao E. Trinidad M. Saffari Doost M. Colognori D. Doudna J. A. (2022). Decorating chromatin for enhanced genome editing using CRISPR-Cas9. Proc. Natl. Acad. Sci. U. S. A. 119 (49), e2204259119. 10.1073/pnas.2204259119 36459645 PMC9894255

[B16] Chen H. F. Chuang H. C. Tan T. H. (2019). Regulation of dual-specificity phosphatase (DUSP) ubiquitination and protein stability. Int. J. Mol. Sci. 20 (11), 2668. 10.3390/ijms20112668 31151270 PMC6600639

[B17] Chen L. Liu S. Tao Y. (2020). Regulating tumor suppressor genes: Post-translational modifications. Signal Transduct. Target Ther. 5 (1), 90. 10.1038/s41392-020-0196-9 32532965 PMC7293209

[B18] Chen X. Kang R. Kroemer G. Tang D. (2021). Broadening horizons: The role of ferroptosis in cancer. Nat. Rev. Clin. Oncol. 18 (5), 280–296. 10.1038/s41571-020-00462-0 33514910

[B19] Chen Z. Ma Y. Guo Z. Song D. Chen Z. Sun M. (2022). Ubiquitin-specific protease 1 acts as an oncogene and promotes lenvatinib efficacy in hepatocellular carcinoma by stabilizing c-kit. Ann. Hepatol. 27 (2), 100669. 10.1016/j.aohep.2022.100669 35045360

[B20] Dai M. Liu M. Yang H. Küçük C. You H. (2022). New insights into epigenetic regulation of resistance to PD-1/PD-L1 blockade cancer immunotherapy: Mechanisms and therapeutic opportunities. Exp. Hematol. Oncol. 11 (1), 101. 10.1186/s40164-022-00356-0 36384676 PMC9667634

[B21] Dashtaki M. E. Ghasemi S. (2022). Anti-angiogenic drug resistance: Roles and targeting of non-coding RNAs (microRNAs and long non-coding RNAs). Curr. Mol. Pharmacol. 16. 10.2174/1874467216666221206100135 36475334

[B22] De Mattia E. Cecchin E. Guardascione M. Foltran L. Di Raimo T. Angelini F. (2019). Pharmacogenetics of the systemic treatment in advanced hepatocellular carcinoma. World J. Gastroenterol. 25 (29), 3870–3896. 10.3748/wjg.v25.i29.3870 31413525 PMC6689804

[B23] di Masi A. Sessa R. L. Cerrato Y. Pastore G. Guantario B. Ambra R. (2022). Unraveling the effects of carotenoids accumulation in human papillary thyroid carcinoma. Antioxidants (Basel) 11 (8), 1463. 10.3390/antiox11081463 36009182 PMC9405418

[B24] Dubbelman A. C. Rosing H. Thijssen B. Gebretensae A. Lucas L. Chen H. (2012). Development and validation of LC-MS/MS assays for the quantification of E7080 and metabolites in various human biological matrices. J. Chromatogr. B Anal. Technol. Biomed. Life Sci. 887-888, 25–34. 10.1016/j.jchromb.2012.01.004 22309776

[B25] Erin N. Grahovac J. Brozovic A. Efferth T. (2020). Tumor microenvironment and epithelial mesenchymal transition as targets to overcome tumor multidrug resistance. Drug Resist Updat 53, 100715. 10.1016/j.drup.2020.100715 32679188

[B26] Fang X. Yan Q. Liu S. Guan X. Y. (2022). Cancer stem cells in hepatocellular carcinoma: Intrinsic and extrinsic molecular mechanisms in stemness regulation. Int. J. Mol. Sci. 23 (20), 12327. 10.3390/ijms232012327 36293184 PMC9604119

[B27] FDA (2016). Lenvatinib in combination with everolimus. Available online: http://www.fda.gov/Drugs/InformationOnDrugs/ApprovedDrugs/ucm501070.htm (Accessed on August 8, 2017).

[B28] Gandhi A. Y. Yu J. Gupta A. Guo T. Iyengar P. Infante R. E. (2022). Cytokine-mediated STAT3 transcription supports ATGL/CGI-58-Dependent adipocyte lipolysis in cancer cachexia. Front. Oncol. 12, 841758. 10.3389/fonc.2022.841758 35785158 PMC9240385

[B29] Gao W. Wang X. Zhou Y. Wang X. Yu Y. (2022). Autophagy, ferroptosis, pyroptosis, and necroptosis in tumor immunotherapy. Signal Transduct. Target Ther. 7 (1), 196. 10.1038/s41392-022-01046-3 35725836 PMC9208265

[B30] Gherardi E. Birchmeier W. Birchmeier C. Vande Woude G. (2012). Targeting MET in cancer: Rationale and progress. Nat. Rev. Cancer 12 (2), 89–103. 10.1038/nrc3205 22270953

[B31] Giammona G. Drago S. E. Calabrese G. Varvarà P. Rizzo M. G. Mauro N. (2022). Galactosylated polymer/gold nanorods nanocomposites for sustained and pulsed chemo-photothermal treatments of hepatocarcinoma. Pharmaceutics 14 (11), 2503. 10.3390/pharmaceutics14112503 36432694 PMC9696514

[B32] Goutal S. Langer O. Auvity S. Andrieux K. Coulon C. Caillé F. (2018). Intravenous infusion for the controlled exposure to the dual ABCB1 and ABCG2 inhibitor elacridar in nonhuman primates. Drug Deliv. Transl. Res. 8 (3), 536–542. 10.1007/s13346-017-0472-6 29294257

[B33] Guo J. Zhao J. Xu Q. Huang D. (2022). Resistance of lenvatinib in hepatocellular carcinoma. Curr. Cancer Drug Targets 22 (11), 865–878. 10.2174/1568009622666220428111327 36267045

[B34] Guo J. Zhu P. Ye Z. Wang M. Yang H. Huang S. (2021). YRDC mediates the resistance of lenvatinib in hepatocarcinoma cells via modulating the translation of KRAS. Front. Pharmacol. 12, 744578. 10.3389/fphar.2021.744578 34658879 PMC8517968

[B35] Guo Y. Wang J. Benedict B. Yang C. van Gemert F. Ma X. (2021). Targeting CDC7 potentiates ATR-CHK1 signaling inhibition through induction of DNA replication stress in liver cancer. Genome Med. 13 (1), 166. 10.1186/s13073-021-00981-0 34663432 PMC8524847

[B36] Guo Y. Xu J. Du Q. Yan Y. Geller D. A. (2021). IRF2 regulates cellular survival and Lenvatinib-sensitivity of hepatocellular carcinoma (HCC) through regulating β-catenin. Transl. Oncol. 14 (6), 101059. 10.1016/j.tranon.2021.101059 33735820 PMC7988337

[B37] Gupta A. Jarzab B. Capdevila J. Shumaker R. Hussein Z. (2016). Population pharmacokinetic analysis of lenvatinib in healthy subjects and patients with cancer. Br. J. Clin. Pharmacol. 81 (6), 1124–1133. 10.1111/bcp.12907 26879594 PMC4876185

[B38] Hao R. Xiang J. Wang B. Chen L. Tan S. (2022).Recent advances in the development of noble metal NPs for cancer therapy. Bioinorg. Chem. Appl. 2022, 2444516. 10.1155/2022/2444516 35126483 PMC8816609

[B39] Hanifeh M. Ataei F. (2022). XIAP as a multifaceted molecule in Cellular Signaling. Apoptosis 27 (7-8), 441–453. 10.1007/s10495-022-01734-z 35661061

[B40] Hardy K. M. Booth B. W. Hendrix M. J. Salomon D. S. Strizzi L. (2010). ErbB/EGF signaling and EMT in mammary development and breast cancer. J. Mammary Gland. Biol. Neoplasia 15 (2), 191–199. 10.1007/s10911-010-9172-2 20369376 PMC2889136

[B41] Hassannia B. Vandenabeele P. Vanden Berghe T. (2019). Targeting ferroptosis to iron out cancer. Cancer Cell 35 (6), 830–849. 10.1016/j.ccell.2019.04.002 31105042

[B42] He X. Hikiba Y. Suzuki Y. Nakamori Y. Kanemaru Y. Sugimori M. (2022). EGFR inhibition reverses resistance to lenvatinib in hepatocellular carcinoma cells. Sci. Rep. 12 (1), 8007. 10.1038/s41598-022-12076-w 35568782 PMC9107466

[B43] Hegde A. Andreev-Drakhlin A. Y. Roszik J. Huang L. Liu S. Hess K. (2020). Responsiveness to immune checkpoint inhibitors versus other systemic therapies in RET-aberrant malignancies. ESMO Open 5 (5), e000799. 10.1136/esmoopen-2020-000799 33097651 PMC7590373

[B44] Hou W. Bridgeman B. Malnassy G. Ding X. Cotler S. J. Dhanarajan A. (2022). Integrin subunit beta 8 contributes to lenvatinib resistance in HCC. Hepatol. Commun. 6 (7), 1786–1802. 10.1002/hep4.1928 35238496 PMC9234648

[B45] Hu B. Zou T. Qin W. Shen X. Su Y. Li J. (2022). Inhibition of EGFR overcomes acquired lenvatinib resistance driven by STAT3-ABCB1 signaling in hepatocellular carcinoma. Cancer Res. 82 (20), 3845–3857. 10.1158/0008-5472.CAN-21-4140 36066408 PMC9574378

[B46] Hu L. Zheng Y. Lin J. Shi X. Wang A. (2022). Comparison of the effects of lenvatinib and sorafenib on survival in patients with advanced hepatocellular carcinoma: A systematic review and meta-analysis. Clin. Res. Hepatol. Gastroenterol. 47, 102061. 10.1016/j.clinre.2022.102061 36473632

[B47] Huang M. Long J. Yao Z. Zhao Y. Zhao Y. Liao J. (2022). METTL1-mediated m7G tRNA modification promotes lenvatinib resistance in hepatocellular carcinoma. Cancer Res. 22, 89–102. 10.1158/0008-5472.CAN-22-0963 36102722

[B48] Huang S. Ma Z. Zhou Q. Wang A. Gong Y. Li Z. (2022). Genome-wide CRISPR/Cas9 library screening identified that DUSP4 deficiency induces lenvatinib resistance in hepatocellular carcinoma. Int. J. Biol. Sci. 18 (11), 4357–4371. 10.7150/ijbs.69969 35864956 PMC9295068

[B49] Huang Z. Xia H. Cui Y. Yam J. W. P. Xu Y. (2023). Ferroptosis: From basic research to clinical therapeutics in hepatocellular carcinoma. J. Clin. Transl. Hepatol. 11 (1), 207–218. 10.14218/JCTH.2022.00255 36406319 PMC9647096

[B50] Ikeda M. Okusaka T. Mitsunaga S. Ueno H. Tamai T. Suzuki T. (2016). Safety and pharmacokinetics of lenvatinib in patients with advanced hepatocellular carcinoma. Clin. Cancer Res. 22 (6), 1385–1394. 10.1158/1078-0432.CCR-15-1354 26500236

[B51] Inc E (2015). Lenvima (lenvatinib) capsules, for oral use: US prescribing information. Available online: http://www.fda.gov (Accessed February 24, 2015).

[B52] Iseda N. Itoh S. Toshida K. Tomiyama T. Morinaga A. Shimokawa M. (2022). Ferroptosis is induced by lenvatinib through fibroblast growth factor receptor-4 inhibition in hepatocellular carcinoma. Cancer Sci. 113 (7), 2272–2287. 10.1111/cas.15378 35466502 PMC9277415

[B53] Ivanisenko N. V. Seyrek K. Hillert-Richter L. K. König C. Espe J. Bose K. (2020). Regulation of extrinsic apoptotic signaling by c-FLIP: Towards targeting cancer networks. Trends Cancer 8 (3), 190–209. 10.1016/j.trecan.2021.12.002 34973957

[B54] Ji J. Wang X. W. (2012). Clinical implications of cancer stem cell biology in hepatocellular carcinoma. Semin. Oncol. 39 (4), 461–472. 10.1053/j.seminoncol.2012.05.011 22846863 PMC3409471

[B55] Jia J. Lunin V. V. Sauvé V. Huang L. W. Matte A. Cygler M. (2002). Crystal structure of the YciO protein from *Escherichia coli* . Proteins 49, 139–141. 10.1002/prot.10178 12211024

[B56] Jia T. Ma Y. Qin F. Han F. Zhang C. (2022). Brain proteome-wide association study linking-genes in multiple sclerosis pathogenesis. Ann. Clin. Transl. Neurol. 10, 58–69. 10.1002/acn3.51699 36475386 PMC9852387

[B57] Jiang H. Li F. Cai L. Chen Q. (2022). Role of the TSPO-NOX4 axis in angiogenesis in glioblastoma. Front. Pharmacol. 13, 1001588. 10.3389/fphar.2022.1001588 36278207 PMC9585329

[B58] Jiang X. Guo S. Zhang Y. Zhao Y. Li X. Jia Y. (2020). LncRNA NEAT1 promotes docetaxel resistance in prostate cancer by regulating ACSL4 via sponging miR-34a-5p and miR-204-5p. Cell Signal 65, 109422. 10.1016/j.cellsig.2019.109422 31672604

[B59] Jin Z. Sun X. Wang Y. Zhou C. Yang H. Zhou S. (2022). Regulation of autophagy fires up the cold tumor microenvironment to improve cancer immunotherapy. Front. Immunol. 13, 1018903. 10.3389/fimmu.2022.1018903 36300110 PMC9589261

[B60] Jindal A. Thadi A. Shailubhai K. (2019). Hepatocellular carcinoma: Etiology and current and future drugs. J. Clin. Exp. Hepatol. 9 (2), 221–232. 10.1016/j.jceh.2019.01.004 31024205 PMC6477125

[B61] Kalinina E. Novichkova M. (2021). Glutathione in protein redox modulation through S-glutathionylation and S-nitrosylation. Molecules 26 (2), 435. 10.3390/molecules26020435 33467703 PMC7838997

[B62] Khan H. Y. Ge J. Nagasaka M. Aboukameel A. Mpilla G. Muqbil I. (2019). Targeting XPO1 and PAK4 in 8505C anaplastic thyroid cancer cells: Putative implications for overcoming lenvatinib therapy resistance. Int. J. Mol. Sci. 21 (1), 237. 10.3390/ijms21010237 31905765 PMC6982268

[B63] Khazaaleh M. Samarasinghe S. Kulasiri D. (2021). A new hierarchical approach to multi-level model abstraction for simplifying ODE models of biological networks and a case study: The G1/S Checkpoint/DNA damage signalling pathways of mammalian cell cycle. Biosystems 203, 104374. 10.1016/j.biosystems.2021.104374 33556446

[B64] Kichi Z. A. Soltani M. Rezaei M. Shirvani-Farsani Z. Rojhannezhad M. (2022). The emerging role of EMT-related lncRNAs in therapy resistance and their applications as biomarkers. Curr. Med. Chem. 29 (26), 4574–4601. 10.2174/0929867329666220329203032 35352644

[B65] Lee Y. S. Kim S. M. Kim B. W. Chang H. J. Kim S. Y. Park C. S. (2018). Anti-cancer effects of HNHA and lenvatinib by the suppression of EMT-mediated drug resistance in cancer stem cells. Neoplasia 20 (2), 197–206. 10.1016/j.neo.2017.12.003 29331886 PMC5767911

[B66] Lei G. Zhuang L. Gan B. (2022). Targeting ferroptosis as a vulnerability in cancer. Nat. Rev. Cancer 22 (7), 381–396. 10.1038/s41568-022-00459-0 35338310 PMC10243716

[B67] Li J. Wang X. Ning C. Wang Z. Wang Y. Zheng M. (2020). Influences of ABC transporter and CYP3A4/5 genetic polymorphisms on the pharmacokinetics of lenvatinib in Chinese healthy subjects. Eur. J. Clin. Pharmacol. 76 (8), 1125–1133. 10.1007/s00228-020-02879-z 32382947

[B68] Li J. Y. Xiao J. Gao M. Zhou H. F. Fan H. Sun F. (2021). IRF/Type I IFN signaling serves as a valuable therapeutic target in the pathogenesis of inflammatory bowel disease. Int. Immunopharmacol. 92, 107350. 10.1016/j.intimp.2020.107350 33444921

[B69] Li Z. Meng X. Wu P. Zha C. Han B. Li L. (2021). Glioblastoma cell-derived lncRNA-containing exosomes induce microglia to produce complement C5, promoting chemotherapy resistance. Cancer Immunol. Res. 9 (12), 1383–1399. 10.1158/2326-6066.CIR-21-0258 34667108

[B70] Liao W. Zhang L. Chen X. Xiang J. Zheng Q. Chen N. (2023). Targeting cancer stem cells and signalling pathways through phytochemicals: A promising approach against colorectal cancer. Phytomedicine 108, 154524. 10.1016/j.phymed.2022.154524 36375238

[B71] Liu C. L. Yang P. S. Wang T. Y. Huang S. Y. Kuo Y. H. Cheng S. P. (2019). PGC1α downregulation and glycolytic phenotype in thyroid cancer. J. Cancer 10 (16), 3819–3829. 10.7150/jca.30018 31333799 PMC6636295

[B72] Liu H. Wang Y. Xue T. Yang Z. Kan S. Hao M. (2023). Roles of m6A modification in oral cancer (Review). Int. J. Oncol. 62 (1), 5. 10.3892/ijo.2022.5453 36382642 PMC9699723

[B73] Liu W. Liu C. Wang H. Xu L. Zhou J. Li S. (2022). Targeting N6-methyladenosine RNA modification combined with immune checkpoint inhibitors: A new approach for cancer therapy. Comput. Struct. Biotechnol. J. 20, 5150–5161. 10.1016/j.csbj.2022.09.017 36187919 PMC9508382

[B74] Liu X. He M. Li L. Wang X. Han S. Zhao J. (2021). EMT and cancer cell stemness associated with chemotherapeutic resistance in esophageal cancer. Front. Oncol. 11, 672222. 10.3389/fonc.2021.672222 34150636 PMC8209423

[B75] Ma X. L. Hu B. Tang W. G. Xie S. H. Ren N. Guo L. (2020). CD73 sustained cancer-stem-cell traits by promoting SOX9 expression and stability in hepatocellular carcinoma. J. Hematol. Oncol. 13 (1), 11. 10.1186/s13045-020-0845-z 32024555 PMC7003355

[B76] Magne T. M. Alencar L. M. R. Carneiro S. V. Fechine L. M. U. D. Fechine P. B. A. Souza P. F. N. (2022). Nano-nutraceuticals for health: Principles and applications. Rev. Bras. Farmacogn. 33, 73–88. 10.1007/s43450-022-00338-7 36466145 PMC9684775

[B77] Malik I. A. Rajput M. Werner R. Fey D. Salehzadeh N. von Arnim C. A. F. (2022). Differential *in vitro* effects of targeted therapeutics in primary human liver cancer: Importance for combined liver cancer. BMC Cancer 22 (1), 1193. 10.1186/s12885-022-10247-6 36402986 PMC9675209

[B78] Maresca L. Stecca B. Carrassa L. (2022). Novel therapeutic approaches with DNA damage response inhibitors for melanoma treatment. Cells 11 (9), 1466. 10.3390/cells11091466 35563772 PMC9099918

[B79] Matuszewski M. Wojciechowski J. Miyauchi K. Gdaniec Z. Wolf W. M. Suzuki T. (2017). A hydantoin isoform of cyclic N6-threonylcarbamoyladenosine (ct6A) is present in tRNAs. Nucleic Acids Res. 45 (4), 2137–2149. 10.1093/nar/gkw1189 27913732 PMC5389693

[B80] Mayani H. Chávez-González A. Vázquez-Santillan K. Contreras J. Guzman M. L. (2022). Cancer stem cells: Biology and therapeutic implications. Arch. Med. Res. 53, 770–784. 10.1016/j.arcmed.2022.11.012 36462951

[B81] McNiel E. A. Tsichlis P. N. (2017). Analyses of publicly available genomics resources define FGF-2-expressing bladder carcinomas as EMT-prone, proliferative tumors with low mutation rates and high expression of CTLA-4, PD-1 and PD-L1. Signal Transduct. Target Ther. 2, 16045. 10.1038/sigtrans.2016.45 28515962 PMC5431749

[B82] Melo-Braga M. N. Ibáñez-Vea M. Kulej K. Larsen M. R. (2021). Comprehensive protocol to simultaneously study protein phosphorylation, acetylation, and N-linked sialylated glycosylation. Methods Mol. Biol. 2261, 55–72. 10.1007/978-1-0716-1186-9_5 33420984

[B83] Mo D. C. Luo P. H. Huang S. X. Wang H. L. Huang J. F. (2021). Safety and efficacy of pembrolizumab plus lenvatinib versus pembrolizumab and lenvatinib monotherapies in cancers: A systematic review. Int. Immunopharmacol. 91, 107281. 10.1016/j.intimp.2020.107281 33338862

[B84] Montagnoli A. Moll J. Colotta F. (2010). Targeting cell division cycle 7 kinase: A new approach for cancer therapy. Clin. Cancer Res. 16 (18), 4503–4508. 10.1158/1078-0432.CCR-10-0185 20647475

[B85] Motzer R. J. Hutson T. E. Glen H. Michaelson M. D. Molina A. Eisen T. (2015). Lenvatinib, everolimus, and the combination in patients with metastatic renal cell carcinoma: A randomised, phase 2, open-label, multicentre trial. Lancet Oncol. 16 (15), 1473–1482. 10.1016/S1470-2045(15)00290-9 26482279

[B86] Muraishi N. Kawamura Y. Akuta N. Shindoh J. Matsumura M. Okubo S. (2022). The impact of lenvatinib on tumor blood vessel shrinkage of hepatocellular carcinoma during treatment: An imaging-based analysis. Oncology 101, 134–144. 10.1159/000526976 36103864 PMC9932824

[B87] Myojin Y. Kodama T. Maesaka K. Motooka D. Sato Y. Tanaka S. (2021). ST6GAL1 is a novel serum biomarker for lenvatinib-susceptible FGF19-driven hepatocellular carcinoma. Clin. Cancer Res. 27 (4), 1150–1161. 10.1158/1078-0432.CCR-20-3382 33288659

[B88] Nakagawa T. Matsushima T. Kawano S. Nakazawa Y. Kato Y. Adachi Y. (2014). Lenvatinib in combination with golvatinib overcomes hepatocyte growth factor pathway-induced resistance to vascular endothelial growth factor receptor inhibitor. Cancer Sci. 105 (6), 723–730. 10.1111/cas.12409 24689876 PMC4317894

[B89] Narita T. Weinert B. T. Choudhary C. (2019). Functions and mechanisms of non-histone protein acetylation. Nat. Rev. Mol. Cell Biol. 20 (3), 156–174. 10.1038/s41580-018-0081-3 30467427

[B90] Nayak D. Paul S. Das C. Bhal S. Kundu C. N. (2022). Quinacrine and Curcumin in combination decreased the breast cancer angiogenesis by modulating ABCG2 via VEGF A. J. Cell Commun. Signal. 10.1007/s12079-022-00692-0 PMC10409692 36326988

[B91] Ning Z. Yang L. Yan X. Wang D. Hua Y. Shi W. (2022). Effect and mechanism of the lenvatinib@H-MnO2-FA drug delivery system in targeting intrahepatic cholangiocarcinoma. Curr. Pharm. Des. 28 (9), 743–750. 10.2174/1381612828666220113161712 35049427

[B92] Omori M. Noro R. Seike M. Matsuda K. Hirao M. Fukuizumi A. (2022). Inhibitors of ABCB1 and ABCG2 overcame resistance to topoisomerase inhibitors in small cell lung cancer. Thorac. Cancer 13 (15), 2142–2151. 10.1111/1759-7714.14527 35719112 PMC9346178

[B93] Ozeki T. Nagahama M. Fujita K. Suzuki A. Sugino K. Ito K. (2019). Influence of CYP3A4/5 and ABC transporter polymorphisms on lenvatinib plasma trough concentrations in Japanese patients with thyroid cancer. Sci. Rep. 9 (1), 5404. 10.1038/s41598-019-41820-y 30931962 PMC6443943

[B94] Paakinaho V. Lempiäinen J. K. Sigismondo G. Niskanen E. A. Malinen M. Jääskeläinen T. (2021). SUMOylation regulates the protein network and chromatin accessibility at glucocorticoid receptor-binding sites. Nucleic Acids Res. 49 (4), 1951–1971. 10.1093/nar/gkab032 33524141 PMC7913686

[B95] Pan G. Liu Y. Shang L. Zhou F. Yang S. (2021). EMT-associated microRNAs and their roles in cancer stemness and drug resistance. Cancer Commun. (Lond) 41 (3), 199–217. 10.1002/cac2.12138 33506604 PMC7968884

[B96] Pan J. Zhang M. Dong L. Ji S. Zhang J. Zhang S. (2022). Genome-Scale CRISPR screen identifies LAPTM5 driving lenvatinib resistance in hepatocellular carcinoma. Autophagy 19, 1184–1198. 10.1080/15548627.2022.2117893 36037300 PMC10012959

[B97] Persano M. Casadei-Gardini A. Burgio V. Scartozzi M. Cascinu S. Rimini M. (2022). Five years of lenvatinib in hepatocellular carcinoma: Are there any predictive and/or prognostic factors? Expert Rev. Anticancer Ther. 23, 19–27. 10.1080/14737140.2023.2156340 36472371

[B98] Pilié P. G. Tang C. Mills G. B. Yap T. A. (2019). State-of-the-art strategies for targeting the DNA damage response in cancer. Nat. Rev. Clin. Oncol. 16 (2), 81–104. 10.1038/s41571-018-0114-z 30356138 PMC8327299

[B99] Primeaux M. Gowrikumar S. Dhawan P. (2022). Role of CD44 isoforms in epithelial-mesenchymal plasticity and metastasis. Clin. Exp. Metastasis 39 (3), 391–406. 10.1007/s10585-022-10146-x 35023031 PMC10042269

[B100] Reisländer T. Groelly F. J. Tarsounas M. (2020). DNA damage and cancer immunotherapy: A sting in the tale. Mol. Cell 80 (1), 21–28. 10.1016/j.molcel.2020.07.026 32810436

[B101] Riccardi G. Bellizzi M. G. Fatuzzo I. Zoccali F. Cavalcanti L. Greco A. (2022). Salivary biomarkers in oral squamous cell carcinoma: A proteomic overview. Proteomes 10 (4), 37. 10.3390/proteomes10040037 36412636 PMC9680331

[B102] Rojas-Prats E. Martinez-Gonzalez L. Gonzalo-Consuegra C. Liachko N. F. Perez C. Ramírez D. (2021). Targeting nuclear protein tdp-43 by cell division cycle kinase 7 inhibitors: A new therapeutic approach for amyotrophic lateral sclerosis. Eur. J. Med. Chem. 210, 112968. 10.1016/j.ejmech.2020.112968 33139113

[B103] Roy Burman D. Das S. Das C. Bhattacharya R. (2021). Alternative splicing modulates cancer aggressiveness: Role in EMT/metastasis and chemoresistance. Mol. Biol. Rep. 48 (1), 897–914. 10.1007/s11033-020-06094-y 33400075

[B104] Sahoo S. Meijles D. N. Pagano P. J. (2016). NADPH oxidases: Key modulators in aging and age-related cardiovascular diseases? Clin. Sci. (Lond). 130 (5), 317–335. 10.1042/CS20150087 26814203 PMC4818578

[B105] Serra V. Wang A. T. Castroviejo-Bermejo M. Polanska U. M. Palafox M. Herencia-Ropero A. (2022). Identification of a molecularly-defined subset of breast and ovarian cancer models that respond to WEE1 or ATR inhibition, overcoming PARP inhibitor resistance. Clin. Cancer Res. 28 (20), 4536–4550. 10.1158/1078-0432.CCR-22-0568 35921524 PMC9561606

[B106] Shetake N. G. Ali M. Kumar A. Bellare J. Pandey B. N. (2022). Theranostic magnetic nanoparticles enhance DNA damage and mitigate doxorubicin-induced cardio-toxicity for effective multi-modal tumor therapy. Biomater. Adv. 142, 213147. 10.1016/j.bioadv.2022.213147 36260957

[B107] ShiB.AnK.WangY.FeiY.GuoC.Cliff ZhangQ. (2022). RNA structural dynamics modulate EGFR-TKI resistance through controlling YRDC translation in NSCLC cells. Genomics Proteomics Bioinforma 2022, S1672–S0229. 10.1016/j.gpb.2022.10.006 36435452

[B108] Shumaker R. Aluri J. Fan J. Martinez G. Ren M. Chen K. (2014). Evaluation of the effects of formulation and food on the pharmacokinetics of lenvatinib (E7080) in healthy volunteers. Int. J. Clin. Pharmacol. Ther. 52 (4), 284–291. 10.5414/CP201937 24548978

[B109] Shumaker R. Aluri J. Fan J. Martinez G. Thompson G. A. Ren M. (2015). Effects of ketoconazole on the pharmacokinetics of lenvatinib (E7080) in healthy participants. Clin. Pharmacol. Drug Dev. 4 (2), 155–160. 10.1002/cpdd.140 26097795 PMC4467237

[B110] Shumaker R. C. Aluri J. Fan J. Martinez G. Thompson G. A. Ren M. (2014). Effect of rifampicin on the pharmacokinetics of lenvatinib in healthy adults. Clin. Drug Investig. 34 (9), 651–659. 10.1007/s40261-014-0217-y PMC4143598 25022720

[B111] Smith A. G. Macleod K. F. (2019). Autophagy, cancer stem cells and drug resistance. J. Pathol. 247 (5), 708–718. 10.1002/path.5222 30570140 PMC6668344

[B112] Smith H. L. Southgate H. Tweddle D. A. Curtin N. J. (2020). DNA damage checkpoint kinases in cancer. Expert Rev. Mol. Med. 22, 22:e2. 10.1017/erm.2020.3 32508294

[B113] Su C. Jin M. Zhang W. (2022). Conservation and diversification of tRNA t6A-modifying enzymes across the three domains of life. Int. J. Mol. Sci. 23 (21), 13600. 10.3390/ijms232113600 36362385 PMC9654439

[B114] Su C. W. Teng W. Lin P. T. Jeng W. J. Chen K. A. Hsieh Y. C. (2022). Similar efficacy and safety between lenvatinib versus atezolizumab plus bevacizumab as the first-line treatment for unresectable hepatocellular carcinoma. Cancer Med. 12, 7077–7089. 10.1002/cam4.5506 36468578 PMC10067067

[B115] Su X. Liu J. Zhang H. Gu Q. Zhou X. Ji M. (2020). Lenvatinib promotes the antitumor effect of doxorubicin in anaplastic thyroid cancer. Onco Targets Ther. 13, 11183–11192. 10.2147/OTT.S278349 33173310 PMC7646435

[B116] Sulkshane P. Teni T. (2022). Myeloid cell leukemia-1: A formidable barrier to anticancer therapeutics and the quest of targeting it. Explor Target Antitumor Ther. 3 (3), 278–296. 10.37349/etat.2022.00083 36045907 PMC9400788

[B117] Sun D. Liu J. Wang Y. Dong J. (2022). Co-administration of MDR1 and BCRP or EGFR/PI3K inhibitors overcomes lenvatinib resistance in hepatocellular carcinoma. Front. Oncol. 12, 944537. 10.3389/fonc.2022.944537 36158676 PMC9496645

[B118] Suryo Rahmanto A. Savov V. Brunner A. Bolin S. Weishaupt H. Malyukova A. (2016). FBW7 suppression leads to SOX9 stabilization and increased malignancy in medulloblastoma. EMBO J. 35 (20), 2192–2212. 10.15252/embj.201693889 27625374 PMC5069553

[B119] Tang P. Dang H. Huang J. Xu T. Yuan P. Hu J. (2018). NADPH oxidase NOX4 is a glycolytic regulator through mROS-HIF1α axis in thyroid carcinomas. Sci. Rep. 8 (1), 15897. 10.1038/s41598-018-34154-8 30367082 PMC6203707

[B120] Tang P. Sheng J. Peng X. Zhang R. Xu T. Hu J. (2022). Targeting NOX4 disrupts the resistance of papillary thyroid carcinoma to chemotherapeutic drugs and lenvatinib. Cell Death Discov. 8 (1), 177. 10.1038/s41420-022-00994-7 35396551 PMC8990679

[B121] Taylor M. H. Leboulleux S. Panaseykin Y. Konda B. de La Fouchardiere C. Hughes B. G. M. (2022). Health-related quality-of-life analyses from a multicenter, randomized, double-blind phase 2 study of patients with differentiated thyroid cancer treated with lenvatinib 18 or 24 mg/day. Cancer Med. 12, 4332–4342. 10.1002/cam4.5308 36464853 PMC9972135

[B122] Tikhonov D. Kulikova L. Rudnev V. Kopylov A. T. Taldaev A. Stepanov A. (2021). Changes in protein structural motifs upon post-translational modification in kidney cancer. Diagn. (Basel) 11 (10), 1836. 10.3390/diagnostics11101836 PMC8534394 34679534

[B123] Toh T. B. Lim J. J. Hooi L. Rashid M. B. M. A. Chow E. K. (2020). Targeting Jak/Stat pathway as a therapeutic strategy against SP/CD44+ tumorigenic cells in Akt/β-catenin-driven hepatocellular carcinoma. J. Hepatol. 72 (1), 104–118. 10.1016/j.jhep.2019.08.035 31541681

[B124] Tsuchida K. Tsujita T. Hayashi M. Ojima A. KelekuLukwete N. Katsuoka F. (2017). Halofuginone enhances the chemo-sensitivity of cancer cells by suppressing NRF2 accumulation. Free Radic. Biol. Med. 103, 236–247. 10.1016/j.freeradbiomed.2016.12.041 28039084

[B125] Uehara Y. Ikeda S. Kim K. H. Lim H. J. Adashek J. J. Persha H. E. (2022). Targeting the FGF/FGFR axis and its co-alteration allies. ESMO Open 7 (6), 100647. 10.1016/j.esmoop.2022.100647 36455506 PMC9808461

[B126] Ullah A. Munir S. Badshah S. L. Khan N. Ghani L. Poulson B. G. (2020). Important flavonoids and their role as a therapeutic agent. Molecules 25 (22), 5243. 10.3390/molecules25225243 33187049 PMC7697716

[B127] Vazhappilly C. G. Hodeify R. Siddiqui S. S. Laham A. J. Menon V. El-Awady R. (2021). Natural compound catechol induces DNA damage, apoptosis, and G1 cell cycle arrest in breast cancer cells. Phytother. Res. 35 (4), 2185–2199. 10.1002/ptr.6970 33289235

[B128] Vimalraj S. (2022). A concise review of VEGF, PDGF, FGF, Notch, angiopoietin, and HGF signalling in tumor angiogenesis with a focus on alternative approaches and future directions. Int. J. Biol. Macromol. 221, 1428–1438. 10.1016/j.ijbiomac.2022.09.129 36122781

[B129] Wang J. T. Zhou J. B. Mao X. L. Zhou L. Chen M. Zhang W. (2022). Commonality and diversity in tRNA substrate recognition in t6A biogenesis by eukaryotic KEOPSs. Nucleic Acids Res. 50 (4), 2223–2239. 10.1093/nar/gkac056 35104889 PMC8887486

[B130] Wang L. Cho K. B. Li Y. Tao G. Xie Z. Guo B. (2019). Long noncoding RNA (lncRNA)-Mediated competing endogenous RNA networks provide novel potential biomarkers and therapeutic targets for colorectal cancer. Int. J. Mol. Sci. 20 (22), 5758. 10.3390/ijms20225758 31744051 PMC6888455

[B131] Wang Y. Tan K. Hu W. Hou Y. Yang G. (2022). LncRNA AC026401.3 interacts with OCT1 to intensify sorafenib and lenvatinib resistance by activating E2F2 signaling in hepatocellular carcinoma. Exp. Cell Res. 420 (1), 113335. 10.1016/j.yexcr.2022.113335 36084669

[B132] Wassermann J. Bagnis C. I. Leenhardt L. Ederhy S. Buffet C. (2022). Pre-therapeutic evaluation and practical management of cardiovascular and renal toxicities in patients with metastatic radioiodine-refractory thyroid cancer treated with lenvatinib. Expert Opin. Drug Saf. 21, 1401–1410. 10.1080/14740338.2022.2153115 36458701

[B133] Weyemi U. Caillou B. Talbot M. Ameziane-El-Hassani R. Lacroix L. Lagent-Chevallier O. (2010). Intracellular expression of reactive oxygen species-generating NADPH oxidase NOX4 in normal and cancer thyroid tissues. Endocr. Relat. Cancer 17 (1), 27–37. 10.1677/ERC-09-0175 19779036

[B134] Wirth L. J. Durante C. Topliss D. J. Winquist E. Robenshtok E. Iwasaki H. (2022). Lenvatinib for the treatment of radioiodine-refractory differentiated thyroid cancer: Treatment optimization for maximum clinical benefit. Oncologist 27 (7), 565–572. 10.1093/oncolo/oyac065 35482606 PMC9256022

[B135] Wirth L. J. Tahara M. Robinson B. Francis S. Brose M. S. Habra M. A. (2018). Treatment-emergent hypertension and efficacy in the phase 3 Study of (E7080) lenvatinib in differentiated cancer of the thyroid (SELECT). Cancer 124 (11), 2365–2372. 10.1002/cncr.31344 29656442

[B136] Wu M. Huang W. Yang N. Liu Y. (2022). Learn from antibody-drug conjugates: Consideration in the future construction of peptide-drug conjugates for cancer therapy. Exp. Hematol. Oncol. 11 (1), 93. 10.1186/s40164-022-00347-1 36348391 PMC9644565

[B137] Xu Q. Li Q. Yang Z. Huang P. Hu H. Mo Z. (2021). Lenvatinib and Cu_2-<i>x</i>_S nanocrystals co-encapsulated in poly(D,L-lactide-*co*-glycolide) for synergistic chemo-photothermal therapy against advanced hepatocellular carcinoma. J. Mater Chem. B 9 (48), 9908–9922. 10.1039/d1tb01808f 34842266

[B138] Xu X. Jiang W. Han P. Zhang J. Tong L. Sun X. (2022). MicroRNA-128-3p mediates lenvatinib resistance of hepatocellular carcinoma cells by downregulating c-met. J. Hepatocell. Carcinoma 9, 113–126. 10.2147/JHC.S349369 35252056 PMC8894104

[B139] Xuan W. Yu H. Zhang X. Song D. (2019). Crosstalk between the lncRNA UCA1 and microRNAs in cancer. FEBS Lett. 593 (15), 1901–1914. 10.1002/1873-3468.13470 31166011

[B140] Xue Z. Vis D. J. Bruna A. Sustic T. van Wageningen S. Batra A. S. (2018). MAP3K1 and MAP2K4 mutations are associated with sensitivity to MEK inhibitors in multiple cancer models. Cell Res. 28 (7), 719–729. 10.1038/s41422-018-0044-4 29795445 PMC6028652

[B141] Yang C. J. Wu M. H. Tsai J. J. Hsu F. T. Hsia T. C. Liu K. C. (2022). Inactivation of AKT/ERK signaling and induction of apoptosis are associated with amentoflavone sensitization of hepatocellular carcinoma to lenvatinib. Anticancer Res. 42 (5), 2495–2505. 10.21873/anticanres.15728 35489726

[B142] Yao J. Ma C. Feng K. Tan G. Wen Q. (2022). Focusing on the role of natural products in overcoming cancer drug resistance: An autophagy-based perspective. Biomolecules 12 (11), 1565. 10.3390/biom12111565 36358919 PMC9687214

[B143] Yarian C. Marszalek M. Sochacka E. Malkiewicz A. Guenther R. Miskiewicz A. (2000). Modified nucleoside dependent Watson-Crick and wobble codon binding by tRNALysUUU species. Biochemistry 39 (44), 13390–13395. 10.1021/bi001302g 11063576

[B144] Ye Z. Wu L. Zhang X. Hu Y. Zheng L. (2021). Quantification of sorafenib, lenvatinib, and apatinib in human plasma for therapeutic drug monitoring by UPLC-MS/MS. J. Pharm. Biomed. Anal. 202, 114161. 10.1016/j.jpba.2021.114161 34052550

[B145] Yi J. W. Park J. Y. Sung J. Y. Kwak S. H. Yu J. Chang J. H. (2015). Genomic evidence of reactive oxygen species elevation in papillary thyroid carcinoma with Hashimoto thyroiditis. Endocr. J. 62 (10), 857–877. 10.1507/endocrj.EJ15-0234 26211532

[B146] Yu T. Yu J. Lu L. Zhang Y. Zhou Y. Zhou Y. (2021). MT1JP-mediated miR-24-3p/BCL2L2 axis promotes Lenvatinib resistance in hepatocellular carcinoma cells by inhibiting apoptosis. Cell Oncol. (Dordr). 44 (4), 821–834. 10.1007/s13402-021-00605-0 33974236 PMC8338827

[B147] Yuan P. Qin H. Y. Wei J. Y. Chen G. Li X. (2022). Proteomics reveals the potential mechanism of Tanshinone IIA in promoting the *ex vivo* expansion of human bone marrow mesenchymal stem cells. Regen. Ther. 21, 560–573. 10.1016/j.reth.2022.11.004 36475023 PMC9700269

[B148] Zhang C. Liu X. Jin S. Chen Y. Guo R. (2022). Ferroptosis in cancer therapy: A novel approach to reversing drug resistance. Mol. Cancer 21 (1), 47. 10.1186/s12943-022-01530-y 35151318 PMC8840702

[B149] Zhang L. Xie H. Wang Y. Wang H. Hu J. Zhang G. (2022). Pharmacodynamic parameters of pharmacokinetic/pharmacodynamic (PK/PD) integration models. Front. Vet. Sci. 9, 860472. 10.3389/fvets.2022.860472 35400105 PMC8989418

[B150] Zhang P. Sun H. Wen P. Wang Y. Cui Y. Wu J. (2021). circRNA circMED27 acts as a prognostic factor and mediator to promote lenvatinib resistance of hepatocellular carcinoma. Mol. Ther. Nucleic Acids 27, 293–303. 10.1016/j.omtn.2021.12.001 35024242 PMC8718824

[B151] Zhang Q. Zheng J. Wang W. Cornett E. M. Kaye A. D. Urits I. (2022). The anticancer effect of metformin combined with epidermal growth factor receptor tyrosine kinase inhibitors in non-small cell lung cancer patients with or without type 2 diabetes mellitus: A systematic review and meta-analysis. Oncol. Ther. 10 (2), 363–375. 10.1007/s40487-022-00209-0 36282467 PMC9681948

[B152] Zhao Y. Zhang Y. N. Wang K. T. Chen L. (2020). Lenvatinib for hepatocellular carcinoma: From preclinical mechanisms to anti-cancer therapy. Biochim. Biophys. Acta Rev. Cancer 1874 (1), 188391. 10.1016/j.bbcan.2020.188391 32659252

[B153] Zheng A. Chevalier N. Calderoni M. Dubuis G. Dormond O. Ziros P. G. (2019). CRISPR/Cas9 genome-wide screening identifies KEAP1 as a sorafenib, lenvatinib, and regorafenib sensitivity gene in hepatocellular carcinoma. Oncotarget 10 (66), 7058–7070. 10.18632/oncotarget.27361 31903165 PMC6925031

[B154] Zittlau K. I. Lechado-Terradas A. Nalpas N. Geisler S. Kahle P. J. Macek B. (2022). Temporal analysis of protein ubiquitylation and phosphorylation during parkin-dependent mitophagy. Mol. Cell Proteomics 21 (2), 100191. 10.1016/j.mcpro.2021.100191 34974192 PMC8808264

[B155] Zouali M. (2014). Transcriptional and metabolic pre-B cell receptor-mediated checkpoints: Implications for autoimmune diseases. Mol. Immunol. 62 (2), 315–320. 10.1016/j.molimm.2014.01.009 24602812

